# Impact of a pharmacist-led implant education service on patient knowledge and healthcare-seeking behaviour: the POSTIMP randomised controlled trial

**DOI:** 10.1016/j.rcsop.2026.100840

**Published:** 2026-09-04

**Authors:** Lionel Tortolano, Nadia Oubaya, Jeremy Hacoon, Muriel Paul, Thomas Brégaint, Laura Benichou, Charles-Henri Flouzat Lachaniette, Romain Bosc, Quentin Misandeau, Valérie Archer

**Affiliations:** a Department of Pharmacy, Henri Mondor Hospital, AP-HP, Créteil, France; b Université Paris-Saclay, Laboratoire Matériaux et Santé, 91400 Orsay, France; c INSERM, IMRB, Université Paris-Est Créteil, F-94010 Créteil, France; d Département de Santé Publique, AP-HP, Hôpital Henri-Mondor, F-94010 Créteil, France; e Department of Orthopaedic Surgery, Henri Mondor Hospital, AP-HP, University of Paris, East Créteil, France; f Cell and Tissue Engineering for Musculoskeletal Disorders (Group 5)/Biology of the NeuroMuscular System (INSERM Team 10)/Mondor Institute for Biomedical Research, Créteil, France; g Department of Plastic, Reconstructive and Maxillofacial Surgery, Henri Mondor Hospital, University Paris XII, 94000 Créteil, France; h Department of Pharmacy, Charles-Foix, AP-HP, Ivry-sur-Seine, France

**Keywords:** Patient education, Medical devices, Implants, Pharmacist, Postoperative care, Randomised controlled trial, Patient safety

## Abstract

**Objective:**

To evaluate a structured pharmacist-led implant education service six months after surgery.

**Methods:**

POSTIMP was a multicentre, participant-blinded randomised controlled trial in orthopaedic and plastic surgery centres within a French university hospital. Adults undergoing hip, knee, or breast implantation were randomised 1:1 to usual support (15-min consultation with implant documents, booklet presentation, and questions) or structured support (30-min interactive consultation adding a six-question quiz, implant image, corrective feedback, and counselling on identification, traceability, follow-up, vigilance, and adverse-event reporting). The primary outcome was correct spontaneous recall of the implant manufacturer, brand, or model at six months, verified against hospital traceability records. Intention-to-treat analyses used logistic regression.

**Results:**

Among 235 participants, spontaneous recall was higher with structured than usual support (13.6% vs 1.7%; odds ratio [OR] 9.02, 95% confidence interval [CI] 2.49–57.9; *p* = 0.004). In an exploratory imputed analysis, correct identification from memory or a predefined list was also higher (29.7% vs 12.8%; OR 2.87, 95% CI 1.49–5.75; *p* = 0.002). Awareness of adverse-event self-reporting increased (OR 2.46, 95% CI 1.40–4.40; *p* = 0.002), and more participants contacted the surgical department (OR 3.12, 95% CI 1.38–7.77; *p* = 0.009), without more emergency visits or hospitalisations.

**Conclusion:**

Structured pharmacist-led implant education improved verified implant knowledge and direct postoperative engagement. It may support earlier, better-directed responses to implant-related concerns, although effects on complications and healthcare use require confirmation.

Trial registration: NCT04302233.

## Highlights

• Structured pharmacist education improved implant recall at six months.
• Implant knowledge and vigilance awareness improved across surgical pathways.
• Patients more often contacted surgical teams after structured education.
• Emergency visits and hospitalisations did not increase.
• Pharmacists can extend postoperative care to implant safety and traceability.

## 1 Introduction

Surgical interventions involving the placement of implantable medical devices (IMDs) became common for patient care across Europe. Approximately 30,000 implants are placed annually at our institution, and this number continues to increase. The long-term use of IMDs may be associated with adverse events. Some are known and anticipated, whereas others are unexpected and may not be identified during preclinical or clinical evaluation before Conformité Européenne (CE) marking, which is the regulatory prerequisite for placing implantable medical devices on the European market and therefore serves a function comparable to marketing authorisation for medicinal products. Post-market surveillance is consequently essential to ensure the continued safety of implanted devices.

Patient education is an established component of implant care and is currently delivered through a range of approaches, including verbal counselling, written information, educational videos and digital resources. However, practices remain heterogeneous across clinical specialties, with substantial variation in the timing, content and delivery of information. Although patients consistently seek information about the characteristics of their implant, the surgical procedure, postoperative recovery, possible complications and recommended follow-up, the information provided is often centred on informed consent and immediate perioperative care.^1^, ^2^, ^3^, ^4^, ^5^, ^6^, ^7^, ^8^, ^9^ Importantly, information being provided does not necessarily mean that patients perceive its relevance, understand it or retain it when it is later needed. Educational interventions have generally improved patients' knowledge, preparedness, satisfaction and understanding of postoperative recommendations, while some studies have also reported reductions in anxiety, opioid consumption or length of hospital stay.^4^, ^7^, ^9^, ^10^, ^11^, ^12^ However, these effects have predominantly been evaluated shortly after the intervention. Evidence regarding the retention of implant-specific information beyond the immediate postoperative period remains limited, and clinical outcomes have been assessed inconsistently. In addition, patients increasingly consult online sources, whose readability, completeness and scientific accuracy vary considerably.^13^, ^14^, ^15^

A particularly important gap concerns the long-term management of implanted devices.^16^ Educational interventions have been evaluated in orthopaedic and other device-related settings but relatively few have addressed patients' ability to identify their specific implant, retain traceability information, understand scheduled follow-up, recognise delayed complications or respond appropriately to a device safety alert^16^, ^17^, ^18^, ^19^. Moreover, most studies have assessed knowledge immediately after education or at hospital discharge, rather than several months after implantation.^17^, ^18^, ^19^, ^20^, ^21^, ^22^, ^23^ To our knowledge, no randomised trial has evaluated spontaneous recall of the patient-specific implant manufacturer or model six months after surgery. In the present study, this six-month assessment was considered a medium-term outcome.

The clinical importance of implant identification and long-term traceability has been particularly evident in the context of breast implant-associated anaplastic large cell lymphoma (BIA-ALCL), for which incomplete implant information has limited the interpretation of safety data.^24^, 25., 26, 27 Studies have shown that a substantial proportion of reported cases lacked sufficient traceability information, partly because patients had not received or retained relevant documentation.^24^, 25. In some cases, implant characteristics could not be retrieved even after explantation due to material degradation, delaying the recognition of associations between implant features and adverse outcomes.^26^, ^27^ The BIA-ALCL experience also highlighted a potential communication gap between information healthcare professionals considered delivered and information patients actually perceived, understood, and retained.

Addressing this communication gap requires more than the passive provision of information. Structured education may support retention by presenting information in accessible language, adapting delivery to the patient's pace, encouraging active processing through questions and immediate corrective feedback, and combining verbal, visual and written formats. Pharmacists are experienced in translating technical healthcare information into practical messages and in checking patients' understanding through interactive counselling. At our institution, pharmacists therefore developed postoperative consultations combining medication counselling with implant-specific education. An initial evaluation showed that this intervention improved patient knowledge at hospital discharge compared with usual pharmaceutical support^7^, ^28^, 29.; however, whether patients could still retrieve this information several months later remained unknown.

The present randomised trial therefore aimed to determine whether structured pharmacist-led postoperative education improved the medium-term retention of patient-specific implant information, assessed six months after surgery. The primary objective was to compare spontaneous recall of the implant manufacturer, brand or model between structured and usual pharmaceutical support. Secondary objectives were to evaluate knowledge of implant characteristics, traceability, follow-up, and adverse-event reporting, as well as safety-related healthcare-seeking behaviours, and to explore whether the intervention effect varied by patient characteristics.

## 2 Methods

### 2.1 Study design

POSTIMP was a prospective, multicentre, parallel-group, participant-blinded randomised controlled superiority trial conducted in two participating surgical centres of different specialties—the orthopaedic surgery department and the plastic surgery department—within the same French university hospital. Participants were recruited between June 2020 and June 2021.The study had initially been scheduled to start in March 2020, but recruitment was postponed during the first French COVID-19 lockdown and began in June 2020 after scheduled surgical activity resumed. Pandemic-related restrictions did not alter the planned content, duration or face-to-face delivery of either pharmaceutical support strategy. Hospital visitation was restricted during this period, which may have increased patient availability for the interviews, although this was not formally assessed. The trial is reported in accordance with the Consolidated Standards of Reporting Trials (CONSORT) statement, and the completed CONSORT checklist is provided as supplementary material.

### 2.2 Ethical considerations

The study was conducted in accordance with the Declaration of Helsinki, the principles of Good Clinical Practice, and French legislation governing clinical research. Ethical approval was granted by the Comité de Protection des Personnes Est I (Dijon, France) at its meeting of 13 February 2020 (SI no. 20.01.20.50011; ID-RCB no. 2019-A03187–50). The favourable opinion was issued on 18 February 2020. The trial was registered at ClinicalTrials.gov (NCT04302233).

Potential participants received both oral and written information about the study and were given at least 24 h to consider participation. They could ask questions before signing by any communication route made available by the clinical or research teams. Written informed consent was obtained before inclusion and before any study-specific data were recorded.

### 2.3 Participants

All patients scheduled for an eligible procedure were systematically considered for participation. Before surgery, the surgeon informed the patient about the study and invited them to participate. During the routine postoperative pharmaceutical interview, the pharmacist was informed that the patient had previously received the study information, answered any further questions, and collected the signed written informed consent form. Baseline data were then recorded in the electronic case-report form (e-CRF). Before randomisation, all participants received routine preoperative medical information from their surgeon during standard surgical consultations. No additional structured implant-specific educational intervention was provided before randomisation as part of the study, and the same usual-care preoperative information pathway applied to both treatment groups.

Adults undergoing scheduled primary implantation or revision of a total hip replacement (THR), total knee replacement (TKR), or breast implant were eligible. Breast implant procedures included reconstruction and augmentation. Participants were required either to speak French or to be accompanied by a French-speaking trusted person. Patients were excluded if they underwent emergency or otherwise unplanned surgery, were unable to communicate in French without a French-speaking trusted person, had a known cognitive or memory disorder, or had previously received a postoperative pharmaceutical interview for an implant placed in the contralateral limb.

### 2.4 Randomisation and blinding

After written informed consent had been obtained, the pharmacist entered the baseline characteristics and all variables required for minimisation into the CleanWeb e-CRF. Participants were then randomised in a 1:1 ratio to new pharmaceutical support (NPS) or usual pharmaceutical support (UPS). Randomisation was performed centrally through the secure web-based CleanWeb system using a minimisation algorithm incorporating a random component to reduce predictability. The prespecified minimisation factors were implant type (breast implant, THR, or TKR), primary implantation or revision at the same anatomical site, age category (<40, 40–65, 65–75, or > 75 years), and educational level (primary or elementary education, secondary education, baccalaureate, or post-baccalaureate education).

Allocation remained concealed until the pharmacist had completed and validated the baseline and minimisation variables in the e-CRF. The system then revealed the allocated group only to the pharmacist responsible for delivering the postoperative intervention. Allocation could not be modified manually. Participants and surgeons remained unaware of the assigned group. Participants knew that the study compared two pharmaceutical support strategies but were not told which strategy represented usual or new support, and the hypothesis favouring either strategy was not disclosed. The pharmacist conducting the 6-month telephone assessment was blinded to treatment allocation and to the identity of the pharmacist who had delivered the postoperative interview.

### 2.5 Intervention rationale and conceptual framework

The intervention was developed pragmatically from a clinical safety concern and from principles of patient education rather than from a single formal behavioural theory. The clinical rationale arose from difficulties encountered during investigations of breast implant-associated anaplastic large-cell lymphoma, in which incomplete identification of implant manufacturers, models, and surface characteristics may have hindered the investigation of associations between implant characteristics and adverse outcomes. During the French regulatory scientific advisory process, patients reported insufficient implant-related information, whereas surgeons reported routinely providing such information. This discrepancy suggested a possible gap between information considered to have been delivered and information actually perceived as important, understood, and retained by patients. These observations informed the choice of outcomes relating to implant identification, traceability, follow-up, and adverse-event reporting.

The structured intervention was based on the assumption that retention would be improved when information was organised, expressed in accessible language, delivered at the patient's pace, and actively processed. Patients were invited to answer questions, reflect on their responses, and receive immediate corrective feedback. Verbal explanations were combined with an implant-specific visual representation to engage both auditory and visual processing. Incorrect or incomplete responses were corrected using the information booklet, thereby reinforcing the association between the information discussed during the consultation and the written material available for later review. The booklet also provided a durable resource that patients could consult after discharge. Pharmacists delivered the intervention because their routine practice includes translating technical healthcare information into practical messages and checking patient understanding through interactive counselling.

### 2.6 Intervention delivery and pharmacist training

Four hospital pharmacists delivered the postoperative interventions. Before study initiation, all four received theoretical training, simulation-based practical training, and supervised workplace training. This workplace training consisted first of observing an experienced pharmacist and then of conducting patient interviews while being observed. Each participant received one individual postoperative consultation in their hospital room between the day after surgery and hospital discharge. The interventions followed predefined materials and educational objectives while allowing pharmacists to respond to individual patient questions. Because the intervention was interactive and adapted to each patient, no formal assessment of strict fidelity to a fixed script was performed.

### 2.7 Interventions

#### 2.7.1 New pharmaceutical support

Participants allocated to NPS received a 30-min structured, interactive consultation. They were provided with an implant identification sheet incorporating a six-question quiz designed to focus attention on implant-related information:

1. Do you know the manufacturer, brand, or model of your implant?
2. In which documents could you find this information?
3. What materials is your implant made of?
4. Which healthcare professionals should be informed that you have an implant?
5. Do you trust the implanted device?
6. To whom can you report an adverse event associated with your implant?

The consultation also included a description of the implant characteristics using a photograph of the participant's implant model; an explanation of medical-device vigilance (referred to as matériovigilance in the French regulatory system) and adverse-event reporting; an explanation of the importance of retaining and consulting the implant identification sheet; and an in-depth presentation of an information booklet specific to knee, hip, or breast implants. The booklet addressed daily life with the implant and the required medical and paramedical follow-up. Each quiz response was discussed, and incorrect or incomplete answers were corrected using the relevant information in the booklet. The orthopaedic booklets had been validated by the hospital Drugs and Medical Devices Committee, and the breast implant booklet had been validated by the French Society of Plastic, Reconstructive and Aesthetic Surgery.

#### 2.7.2 Usual pharmaceutical support

Participants allocated to UPS received a 15-min consultation. They were provided with the same implant identification sheet and the same implant-specific booklet as the NPS group. The booklet was presented orally, and the pharmacist answered any questions raised by the patient. UPS did not include the structured six-question quiz, the implant-specific photographic presentation, systematic corrective feedback, or the detailed structured discussion of implant identification and medical-device vigilance used in NPS. In both groups, participants were advised to contact the surgical department if implant-related questions arose after discharge and were cautioned that information obtained from unverified online sources could be inaccurate or outdated. The components of the two pharmaceutical support strategies are summarised in Table 1.

**Table 1:** comparison of the two pharmaceutical support strategies.

Component	Usual pharmaceutical support	New pharmaceutical support
Format	Individual consultation in the patient's room	Individual structured consultation in the patient's room
Timing	Between the day after surgery and discharge	Between the day after surgery and discharge
Frequency	One postoperative consultation	One postoperative consultation
Planned duration	15 min	30 min
Implant identification sheet	Provided	Provided and used as an active educational tool
Six-question quiz	No	Yes
Implant-specific photograph	No	Yes
Implant characteristics and materials	No structured additional discussion	Detailed structured discussion
Implant-specific booklet	Provided and presented orally	Provided and presented in depth
Medical-device vigilance and adverse-event reporting	No structured presentation	Structured presentation
Corrective feedback	No structured procedure	Immediate feedback using the booklet
Patient questions	Answered	Answered throughout the interactive consultation

### 2.8 Six-month telephone follow-up

During the postoperative consultation, participants were asked to indicate their preferred day, time, and telephone number for the 6-month follow-up call. At 6 months, all participants were contacted by a pharmacist who was blinded to treatment allocation and to the identity of the pharmacist who had conducted the postoperative consultation. The telephone interview followed the same standardised questionnaire for both groups, was recorded directly in the CleanWeb e-CRF, and was expected to last approximately 20 min.

The 6-month questionnaire, including all study outcomes, had been defined before recruitment and was included in the documents reviewed by the ethics committee. The call concluded with a reminder to all participants of the importance of the implant-related information discussed during the interview and of retaining their implant documentation. This reminder served as reinforcement for participants in the NPS group and as catch-up education for participants in the UPS group.

### 2.9 Outcomes

All primary and secondary outcomes were defined before study initiation and were included in the protocol and assessment documents reviewed by the ethics committee.

#### 2.9.1 Primary outcome

The prespecified primary outcome was correct spontaneous recall, without prompting, of the manufacturer, brand or model of the participant's implant six months after surgery. The response was compared with the patient-specific information recorded in the hospital implant traceability record and coded as correct or incorrect.

After recruitment had begun, an additional list-assisted recognition item was introduced for participants who could not provide a correct spontaneous response. These participants were presented with a predefined list of manufacturers, brands, or model names. Because this item was introduced after the first participants had already completed their assessments, it was not available for the entire study population. It was therefore analysed separately as an additional exploratory outcome and was not part of the prespecified primary outcome.

#### 2.9.2 Secondary outcomes

Secondary outcomes were grouped into five domains:

• Implant identification and traceability: reported receipt of the implant card or traceability form; ability to access and read the batch number for THR or TKR or the serial number for a breast implant; and recall of the implantation date and surgeon's name.
• Implant characteristics: knowledge of implant materials; breast implant surface texturing; and, for THR, knowledge of the surgical approach and whether fixation was cemented or uncemented.
• Follow-up and medical-device vigilance: knowledge of the implantation centre, the need for regular medical follow-up, the possibility of self-reporting an implant-related adverse event, and the persons or institutions to whom an implant-related problem could be reported.
• Patient-reported experience: satisfaction with the information provided during the pharmaceutical consultation and confidence in the implant.
• Healthcare-seeking behaviour: calls to the surgical department, unplanned implant-related consultations outside the study hospital, emergency department visits, and hospitalisations related to the implant during the 6-month follow-up period.

For traceability outcomes, participants were asked to read the relevant batch or serial number directly from their implant card or traceability form during the telephone interview. A correctly reported identifier was therefore taken as evidence that the participant had access to the document at the time of assessment.

All participants were assessed using identical question wording and response coding. Outcomes were analysed individually and were coded as binary variables, except for satisfaction and confidence, which were recorded using Likert-type response categories. No composite knowledge score was calculated. The questionnaire assessed discrete factual and actionable items rather than an underlying psychometric construct; consequently, internal-consistency measures such as Cronbach's alpha were not applicable.

### 2.10 Sample-size calculation

The sample-size calculation was based on the primary outcome, namely the ability to recall the manufacturer, brand, or model of the implant at 6 months. In the absence of published data directly estimating spontaneous recall of the patient-specific implant manufacturer, brand or model at six months, the assumptions used for the sample-size calculation were defined during protocol development based on the available literature and preliminary observations. A total of 176 analysable participants (88 per group) were required to provide 80% power, with a two-sided type I error rate of 5%, to detect a clinically meaningful absolute difference of 15 percentage points between the UPS and NPS groups. Allowing for 25% attrition or unavailable 6-month assessments, the planned recruitment target was 236 participants. The trial was powered for the primary outcome rather than for secondary outcomes or subgroup analyses.

### 2.11 Statistical analysis

Baseline characteristics were summarised by treatment group using the mean and standard deviation or the median and interquartile range, as appropriate, for continuous variables, and counts and percentages for categorical variables. Analyses were performed in the intention-to-treat (ITT) population, which included all randomised participants in their assigned group. Outcomes missing due to the 6-month assessment being unavailable were imputed using the missForest package.

Primary and secondary binary outcomes were analysed using logistic regression models. Firth's penalised logistic regression was used for outcomes with rare events. Effect estimates are reported as odds ratios with 95% confidence intervals. Sensitivity analyses included a per-protocol analysis and analyses adjusted for the prespecified minimisation factors: implant type, primary implantation or revision, age category, and educational level. The per-protocol population consisted of participants who completed the 6-month assessment and, in the NPS group, received a postoperative pharmaceutical consultation lasting at least 20 min.

Prespecified subgroup analyses evaluated whether the intervention effect differed according to implant type (breast implant vs THR/TKR), age (<65 vs ≥65 years), primary implantation or revision, healthcare professional status, educational level (elementary or middle school vs high school or higher), and, among breast implant recipients, augmentation or reconstruction. For each subgroup analysis, an interaction term between treatment allocation and the subgroup variable was included in the logistic regression model, and the interaction *p*-value was reported. Subgroup analyses were considered exploratory and were interpreted cautiously.

All tests were two-sided, and statistical significance was defined as *p* < 0.05. Analyses were performed using R software (R Core Team, 2024; R Foundation for Statistical Computing, Vienna, Austria) https://www.R-project.org/.

## 3 Results

### 3.1 Participant flow and baseline characteristics

**Fig. 1:**
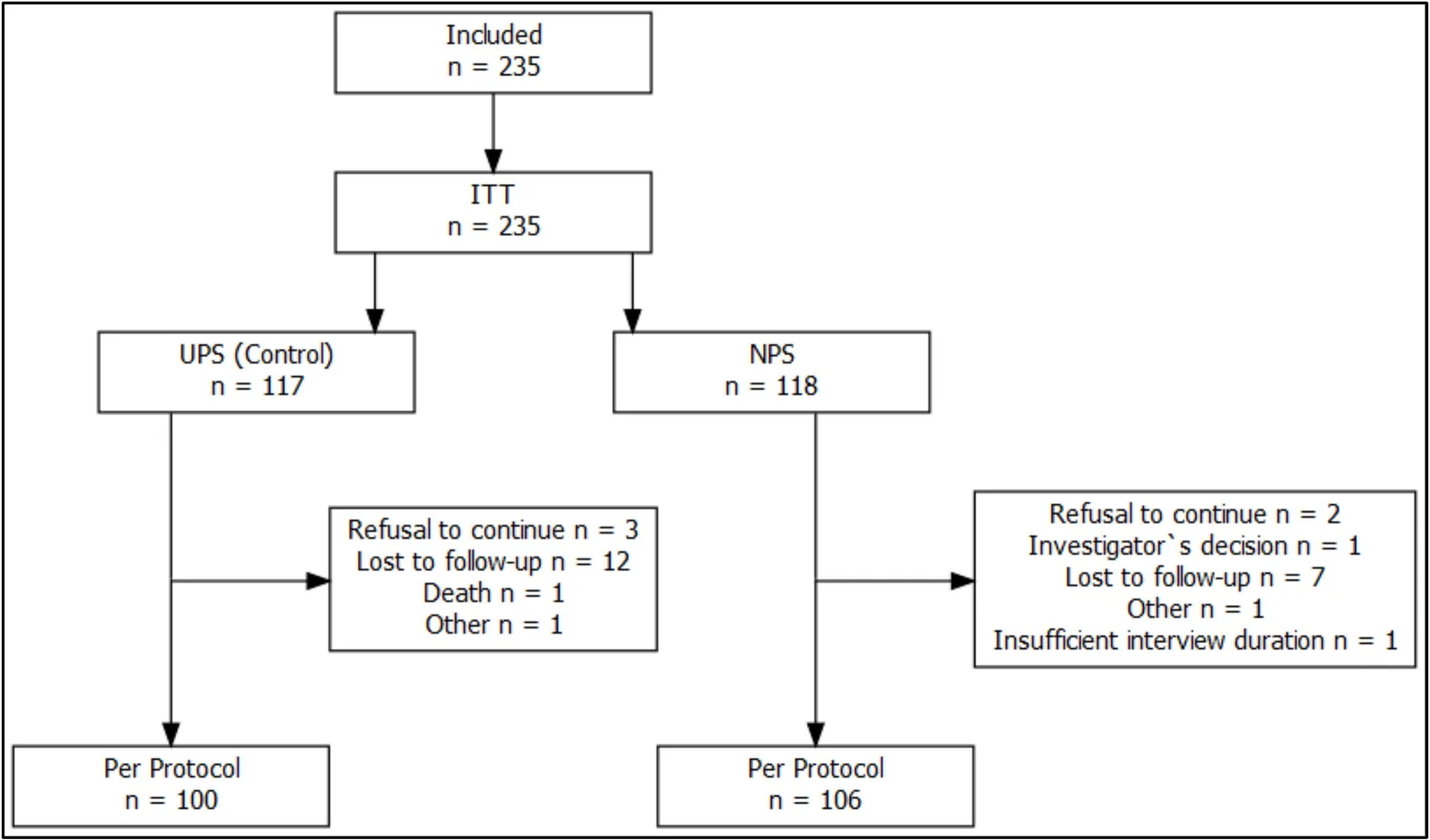
CONSORT flow diagram of participants through the trial.

**Fig. 2:**
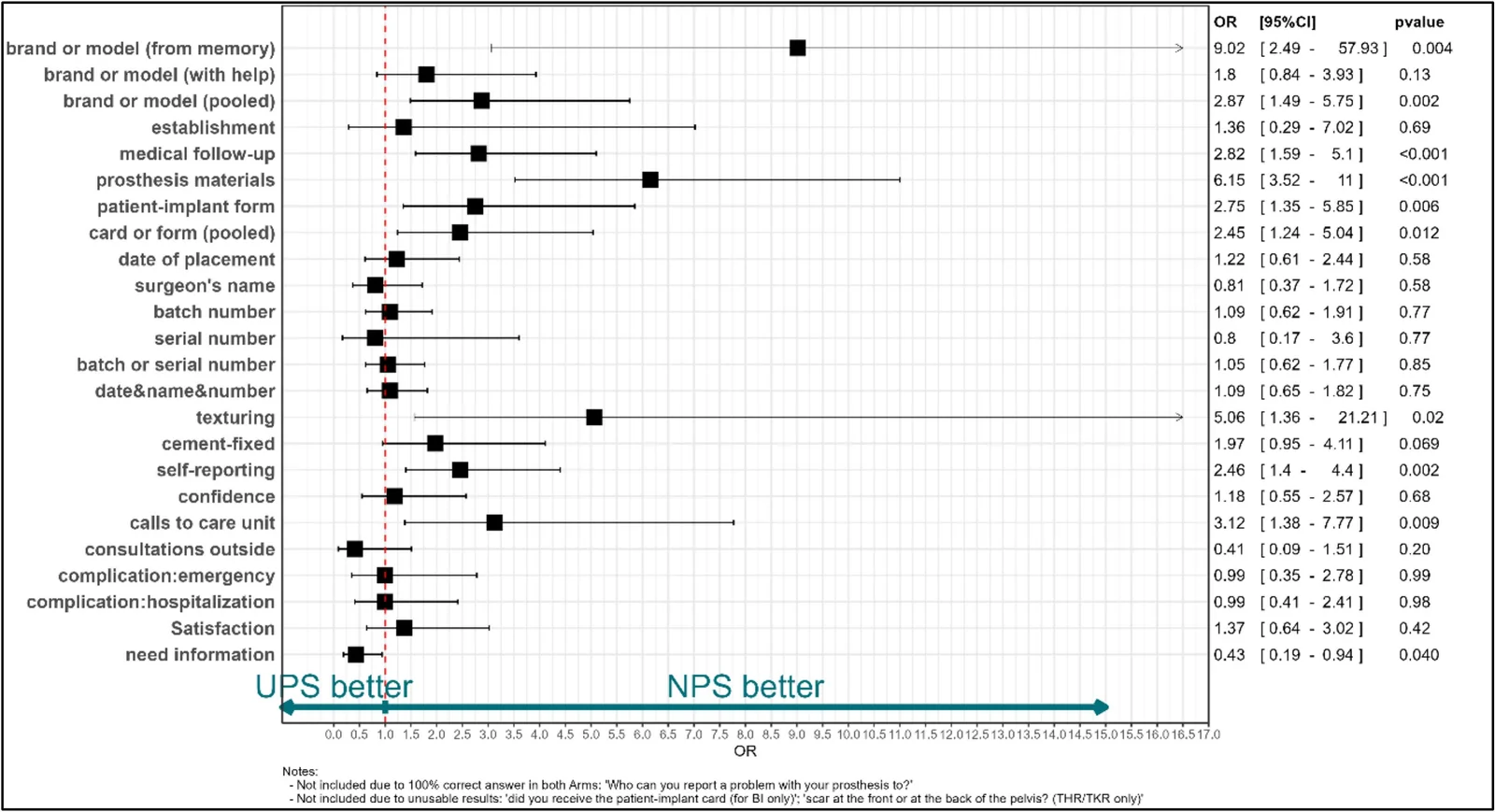
Unadjusted odds ratios for primary and secondary outcomes in the intention-to-treat population. Odds ratios greater than 1 indicate that the outcome was more frequent in the NPS group. Whether a higher frequency represents a favourable result depends on the definition and coding of the individual outcome. UPS better means patients in the UPS group responded “yes” or gave the right answer more often than patients in the NPS group.

Between June 2020 and June 2021, 235 participants provided written informed consent and were randomised: 117 to usual pharmaceutical support (UPS) and 118 to new pharmaceutical support (NPS) (Fig. 1). The per-protocol population comprised 100 participants in the UPS group and 106 in the NPS group. Baseline characteristics according to the availability of the primary outcome are presented in Table S1. (See Fig. 2.)

Overall, 140 participants (59.6%) were women, and the mean age was approximately 59 years. A total of 196 participants underwent total hip or knee replacement and 39 underwent breast implantation. Primary implantation accounted for 205 procedures (87.2%), and 34 participants (14.5%) were healthcare professionals. The treatment groups were descriptively balanced with respect to the prespecified minimisation factors and the main baseline characteristics (Table 2).

**Table 2:** Baseline characteristics of patients in the intention-to-treat population.

Characteristic	UPS	NPS
	*N* = 117^1^	*N* = 118^1^
Centre		
Orthopaedic surgery	98 (83.8%)	98 (83.1%)
Plastic surgery	19 (16.2%)	20 (16.9%)
Sex		
	52 (44.4%)	43 (36.4%)
Female	65 (55.6%)	75 (63.6%)
Age	59.3 (17.3)	58.7 (17.0)
Level of study		
Elementary school/study certificate	27 (23.1%)	30 (25.4%)
Middle school diploma	23 (19.7%)	23 (19.5%)
High school diploma	19 (16.2%)	16 (13.6%)
Post-high school diploma	48 (41.0%)	49 (41.5%)
French speaking	116 (99.1%)	118 (100.0%)
Experience as a health care professional	17 (14.5%)	17 (14.4%)
Prosthesis type		
THR	60 (51.3%)	60 (50.8%)
TKR	38 (32.5%)	38 (32.2%)
Breast implant	19 (16.2%)	20 (16.9%)
Primo-implantation/resumption		
Primary implantation	102 (87.2%)	103 (87.3%)
Resumption	15 (12.8%)	15 (12.7%)
Brand/manufacturer of the breast implant, N_UPS_ = 19, N_NPS_ = 20		
Sebbin	17 (89.5%)	18 (90.0%)
Motiva	2 (10.5%)	2 (10.0%)
Brand/manufacturer of the prosthesis (THR/TKR), N_UPS_ = 98, N_NPS_ = 98		
Medacta	42 (42.9%)	34 (34.7%)
Osteal medical (=ceraver)	37 (37.8%)	40 (40.8%)
Groupe Lepine	9 (9.2%)	10 (10.2%)
SYMBIOS	10 (10.2%)	11 (11.2%)
Depuy Synthes (Johnson & Johnson)	0 (0.0%)	1 (1.0%)
Medicalex	0 (0.0%)	1 (1.0%)
STANMORE STRYKER	0 (0.0%)	1 (1.0%)
Indication (for breast implants)		
Breast augmentation surgery	9 (47.4%)	14 (70.0%)
Breast reconstruction surgery	10 (52.6%)	6 (30.0%)
Indication for the placement of the prosthesis (THR)		
Osteoarthritis	35 (58.3%)	37 (61.7%)
Aseptic osteonecrosis	22 (36.7%)	17 (28.3%)
Dysplasia	1 (1.7%)	3 (5.0%)
Other	2 (3.3%)	3 (5.0%)
Indication for the placement of the prosthesis (TKR)		
Osteoarthritis	38 (100.0%)	34 (89.5%)
Other	0 (0.0%)	4 (10.5%)

### 3.2 Intervention delivery and analysis populations

All 118 participants allocated to NPS began the allocated intervention. One consultation was discontinued before the prespecified minimum duration of 20 min. This participant remained in the intention-to-treat analysis but was excluded from the per-protocol analysis.

### 3.3 Primary outcome

At six months, 16 of 118 participants allocated to NPS (13.6%) correctly recalled the manufacturer, brand or model of their implant without prompting, compared with 2 of 117 participants allocated to UPS (1.7%). The absolute between-group difference was 11.8 percentage points, corresponding to an odds ratio of 9.02 (95% CI 2.49–57.9; *p* = 0.004). Adjustment for the prespecified minimisation factors produced a similar effect estimate (adjusted OR 8.84, 95% CI 2.48–48.4; *p* < 0.001).

The list-assisted recognition item was introduced after recruitment had begun and was therefore considered exploratory. In the imputed intention-to-treat analysis, list-assisted recognition among participants who did not spontaneously recall their implant did not differ significantly between groups (UPS: 13/115 [11.3%] vs NPS: 19/102 [18.6%]; OR 1.80, 95% CI 0.84–3.93; *p* = 0.132). When spontaneous recall and list-assisted recognition were combined, correct identification was higher with NPS than with UPS (35/118 [29.7%] vs 15/117 [12.8%]; OR 2.87, 95% CI 1.49–5.75; *p* = 0.002).

Participants allocated to NPS were more likely than those allocated to UPS to know that regular medical follow-up was required (94/118 [79.7%] vs 68/117 [58.1%]; OR 2.82, 95% CI 1.59–5.10; *p* = 0.00045) and to correctly identify the materials of their implant (79/118 [66.9%] vs 29/117 [24.8%]; OR 6.15, 95% CI 3.52–11.0; *p* = 3.85 × 10^−10^).

Participants allocated to NPS were also more likely to report having access to an implant traceability document (104/118 [88.1%] vs 88/117 [75.2%]; OR 2.45, 95% CI 1.24–5.04; *p* = 0.012) and to know that they could self-report an implant-related adverse event (92/118 [78.0%] vs 69/117 [59.0%]; OR 2.46, 95% CI 1.40–4.40; *p* = 0.002).

### 3.4 Secondary implant knowledge and safety-related outcomes

No significant differences were observed between groups for recall of the date of implantation, the surgeon's name, or implant identifiers (batch or serial number), nor for knowledge of the appropriate institutions or professionals to contact in case of a prosthesis-related issue. Notably, all patients in both groups correctly identified the appropriate reporting contacts, and more than 80% correctly recalled the implantation date and surgeon's name.

Among patients with total hip replacement, correct identification of scar location was higher in the NPS group than in the UPS group (100% vs 91.7%; OR 12.0, 95% CI 1.31–1588). Knowledge of whether the prosthesis was cemented was also higher in the NPS group (55.0% vs 38.3%; OR 1.97, 95% CI 0.95–4.11), although this difference did not reach statistical significance (*p* = 0.069).

### 3.5 Patient-reported experience and healthcare-seeking behaviour

Overall, patients in the NPS group were more likely to contact the hospital ward after discharge than those in the UPS group (OR 3.12, 95% CI 1.38–7.77; *p* = 0.009). This difference was primarily driven by patients with breast implants, whereas no significant difference was observed among those with orthopaedic implants. In contrast, the proportion of patients who had a consultation, emergency department visit, or hospitalisation related to the prosthesis did not differ between groups.

High levels of satisfaction with the pharmaceutical support were reported in both groups, with no significant difference (UPS: 85.5% vs NPS: 89.0%). Similarly, most patients reported being totally or somewhat confident in their prosthesis (UPS: 86.3% vs NPS: 88.1%), with no difference between groups.

Patients in the NPS group were less likely to seek additional information about their convalescence or long-term postoperative outcomes (OR 0.43, 95% CI 0.19–0.94; *p* = 0.04).

The estimated effect of NPS on the primary outcome remained directionally consistent in the per-protocol analysis and after adjustment for the prespecified minimisation factors. The corresponding odds ratios, 95% confidence intervals and exact *p*-values are reported in Tables S2–S4. Prespecified subgroup analyses evaluated possible effect modification by implant type, age, educational level, primary implantation versus revision, healthcare professional status and, among breast implant recipients, augmentation versus reconstruction. Complete unadjusted estimates for the prespecified primary, exploratory and secondary outcomes in the intention-to-treat population are presented in Table 3.

**Table 3:** Prespecified primary, exploratory and secondary outcomes in the intention-to-treat population.

Characteristic	N	UPS, N = 117^1^	NPS, N = 118^1^	OR	CI	*p*-value
Correct answer: manufacturer/brand or model (from memory)	235	2 (1.7%) N = 117	16 (13.6%) N = 118	9.02	2.49–57.9	**0.004**
Correct answer: manufacturer/brand or model (with the help of a list among those who failed from memory)^2^	217	13 (11.3%) *N* = 115	19 (18.6%) *N* = 102	1.80	0.840–3.93	0.132
Correct answer: manufacturer/brand or model (from memory or with the help of a list)^2^	235	15 (12.8%) *N* = 117	35 (29.7%) *N* = 118	2.87	1.49–5.75	**0.002**
Correct answer: establishment of the prosthesis placement	235	113 (96.6%) N = 117	115 (97.5%) N = 118	1.36	0.290–7.02	0.694
Correct answer: regular medical follow-up required?	235	68 (58.1%) N = 117	94 (79.7%) N = 118	2.82	1.59–5.10	**<0.001**
Correct answer: prosthesis materials	235	29 (24.8%) N = 117	79 (66.9%) N = 118	6.15	3.52–11.0	**<0.001**
Correct answer: Did you receive the patient-implant card (BI only)	39	19 (100.0%) *N* = 19	19 (95.0%) *N* = 20	0.330	0.000–6.65	**0.480**
Correct answer: Did you receive the patient-implant form (THR/TKR only)	196	69 (70.4%) *N* = 98	85 (86.7%) N = 98	2.75	1.35–5.85	**0.006**
Correct answer: Did you receive the patient-implant card or form (pooled)	235	88 (75.2%) *N* = 117	104 (88.1%) *N* = 118	2.45	1.24–5.04	**0.012**
Correct answer: date of prosthesis placement	235	96 (82.1%) N = 117	100 (84.7%) N = 118	1.22	0.610–2.44	0.579
Correct answer: surgeon's name	235	103 (88.0%) N = 117	101 (85.6%) N = 118	0.810	0.370–1.72	0.581
Correct answer: prosthesis batch number (THR/TKR)	196	54 (55.1%) *N* = 98	56 (57.1%) N = 98	1.09	0.620–1.91	0.773
Correct answer: breast implant serial number	39	15 (78.9%) *N* = 19	15 (75.0%) *N* = 20	0.800	0.170–3.60	0.770
Correct answer: batch or serial number (THR/TKR or BI)	235	69 (59.0%) *N* = 117	71 (60.2%) *N* = 118	1.05	0.620–1.77	0.852
Correct answer to the 3 items: date of prosthesis placement, surgeon's name, batch or serial number	235	61 (52.1%) N = 117	64 (54.2%) N = 118	1.09	0.650–1.82	0.747
Correct answer: texturing of your prosthesis surface (BI only)	39	6 (31.6%) *N* = 19	14 (70.0%) *N* = 20	5.06	1.36–21.2	**0.020**
Correct answer: scar at the front or at the back of the pelvis? (THR only)	120	55 (91.7%) *N* = 60	60 (100.0%) N = 60	12.0	1.31–1588	**0.024**
Correct answer: fixed prosthesis with or without cement? (THR only)	120	23 (38.3%) N = 60	33 (55.0%) N = 60	1.97	0.950–4.11	0.069
Knowledge of the possibility of self-reporting an adverse event associated with the prosthesis	235	69 (59.0%) *N* = 117	92 (78.0%) *N* = 118	2.46	1.40–4.40	**0.002**
Correct answer: To whom can you report a problem with your prosthesis to?	235			1.00	NA-NA	not estimable^3^
yes		117 (100.0%) N = 117	118 (100.0%) N = 118			
Level of confidence in the prosthesis (categorical from Likert scale)	235			1.18	0.550–2.57	0.678
Neutral to not confident at all		16 (13.7%) N = 117	14 (11.9%) N = 118			
Totally or somewhat confident		101 (86.3%) N = 117	104 (88.1%) N = 118			
Number of calls to the care unit where the patient was hospitalised after the prosthesis was placed	235			3.12	1.38–7.77	**0.009**
0		109 (93.2%) N = 117	96 (81.4%) N = 118			
At least 1		8 (6.8%) N = 117	22 (18.6%) N = 118			
Number of consultations related to the prosthesis with a surgeon outside the hospital Henri Mondor	235			0.410	0.090–1.51	0.205
0		110 (94.0%) N = 117	115 (97.5%) N = 118			
At least 1		7 (6.0%) N = 117	3 (2.5%) N = 118			
Admission to the emergency room for a complication related to the prosthesis	235	8 (6.8%) N = 117	8 (6.8%) N = 118	0.990	0.350–2.78	0.986
Hospitalisation for a complication related to the prosthesis	235	11 (9.4%) N = 117	11 (9.3%) N = 118	0.990	0.410–2.41	0.983
Satisfaction with the information provided about the prosthesis after the operation (categorical from Likert scale)	235			1.37	0.640–3.02	0.421
Quite not satisfied		17 (14.5%) N = 117	13 (11.0%) N = 118			
Satisfied		100 (85.5%) N = 117	105 (89.0%) N = 118			
Do you need information about your implant or your convalescence? 2	235	107 (91.5%) N = 117	97 (82.2%) N = 118	0.430	0.190–0.940	**0.040**

1 n (%) N=N.

2 List-assisted recognition was introduced after recruitment had begun and was therefore considered exploratory. Before imputation, this item was administered to 60 participants in the UPS group and 51 in the NPS group. The item concerning the need for additional information was also available for only part of the study population.3 The effect could not be estimated because all participants answered correctly.

### 3.6 Stratified analyses

Prespecified subgroup analyses were conducted to explore whether the effect of NPS varied according to implant type, age, primary implantation or revision, healthcare professional status, educational level and, among breast implant recipients, augmentation or reconstruction. These analyses were not powered to demonstrate treatment effects within individual subgroups and were therefore interpreted as exploratory, particularly when subgroup sizes were small or confidence intervals were wide.

No evidence of interaction between treatment allocation and implant type was observed for the primary outcome of spontaneous recall of the implant manufacturer, brand or model (p for interaction = 0.80). This finding indicates that no heterogeneity of the intervention effect on the primary outcome was detected between breast and orthopaedic implant recipients.

For knowledge of implant materials, the estimated effect of NPS was greater among breast implant recipients (OR 41.2, 95% CI 6.31–832; *p* = 0.001) than among participants with total hip or knee replacements (OR 5.15, 95% CI 2.81–9.71; *p* < 0.001). The interaction did not reach the conventional threshold for statistical significance (p for interaction = 0.08), but the difference in effect estimates suggests possible heterogeneity that warrants further investigation. Independently of treatment allocation, correct reporting of the breast implant serial number was more frequent than correct reporting of the batch number of an orthopaedic implant: 78.9% and 75.0% of breast implant recipients in the UPS and NPS groups, respectively, compared with 55.1% and 57.1% of orthopaedic implant recipients.

Age was associated with heterogeneity in the effect of NPS on knowledge of implant materials. The estimated effect was greater among participants aged <65 years (OR 13.1, 95% CI 5.93–31.0; *p* < 0.001) than among those aged ≥65 years (OR 2.60, 95% CI 1.16–6.00; *p* = 0.022), with evidence of interaction (p for interaction = 0.006). For implant-related consultations outside the study hospital, NPS was associated with fewer consultations among participants aged <65 years (OR 0.08, 95% CI 0.00–0.74; *p* = 0.022), whereas no association was observed among those aged ≥65 years (OR 1.46, 95% CI 0.27–9.10; *p* = 0.70; p for interaction = 0.046) (Table S6). These findings suggest that age may influence which dimensions of implant-related knowledge or healthcare-seeking behaviour are affected by the intervention, but they require confirmation in larger samples.

No evidence of interaction was observed according to the indication for breast implantation—augmentation or reconstruction—or for most outcomes according to primary implantation versus revision surgery. For implant-related hospitalisation, the subgroup estimates differed between participants undergoing revision (OR 1.72, 95% CI 0.61–5.23; *p* = 0.30) and those undergoing primary implantation (OR 0.14, 95% CI 0.01–1.07; *p* = 0.10), with a borderline interaction (p for interaction = 0.05) (Tables S7–S8). Because neither within-subgroup estimate was statistically significant and both confidence intervals were wide, the clinical interpretation of this interaction remains uncertain.

Educational level was also associated with heterogeneity in selected outcomes. NPS had a greater estimated effect on knowledge of follow-up requirements among participants with elementary- or middle-school education (OR 5.47, 95% CI 2.32–13.8; *p* < 0.001) than among participants with high-school or higher education (OR 1.66, 95% CI 0.76–3.71; *p* = 0.20; p for interaction = 0.049). Conversely, NPS was associated with fewer implant-related consultations outside the study hospital among participants with higher educational attainment (OR 0.07, 95% CI 0.00–0.63; *p* = 0.01), whereas no association was observed among those with lower educational attainment (OR 2.29, 95% CI 0.36–24.2; *p* = 0.40; p for interaction = 0.02) (Table S9).

Healthcare professional status was associated with heterogeneity in the exploratory list-assisted recognition outcome. Among healthcare professionals, NPS was associated with greater recognition of the manufacturer, brand or model from a predefined list when spontaneous recall was unsuccessful (OR 16.7, 95% CI 1.64–22.78; *p* = 0.01), whereas no association was observed among participants who were not healthcare professionals (OR 1.27, 95% CI 0.56–2.86; *p* = 0.60; p for interaction = 0.04). When spontaneous recall and list-assisted recognition were combined, the estimated effect remained greater among healthcare professionals (OR 19.8, 95% CI 2.01–26.74; *p* = 0.02) than among other participants (OR 2.24, 95% CI 1.14–4.56; *p* = 0.007), although evidence of interaction was less conclusive (p for interaction = 0.08). A similar pattern was observed for awareness of adverse-event self-reporting (Table S10). Because list-assisted recognition was introduced after recruitment had begun, these subgroup findings should be regarded as particularly exploratory.

Taken together, these analyses suggest that the effect of structured pharmaceutical support may differ according to the type of information assessed and certain patient characteristics. In particular, age, educational level and healthcare professional status may influence how patients retain or use implant-related information. However, these subgroup results should be considered hypothesis-generating and should not currently be used to restrict the intervention to specific patient groups. Their main value is to identify potential factors to examine prospectively when adapting and evaluating the service in larger and more diverse populations.

## 4 Discussion

### 4.1 Principal findings and clinical interpretation

This multicentre randomised controlled trial showed that a structured pharmacist-led implant education service produced a coherent pattern of improvements six months after surgery. Compared with usual pharmaceutical support, NPS increased spontaneous recall of the implant manufacturer, brand or model, knowledge of implant materials and follow-up requirements, awareness of implant traceability documentation and knowledge of the possibility of self-reporting an implant-related adverse event. Participants allocated to NPS were also more likely to contact the surgical department after discharge, without a corresponding increase in emergency department attendance or hospitalisation.

These findings suggest that the intervention influenced both information retention and postoperative pathway navigation. The higher frequency of direct contact with the surgical department, without a corresponding increase in emergency attendance or hospitalisation, is compatible with more targeted information-seeking behaviour. However, because the reasons and consequences of these contacts were not recorded, whether earlier contact can prevent avoidable escalation remains hypothesis-generating and requires confirmation in studies powered for clinical outcomes.

Correct spontaneous recall of the implant manufacturer, brand or model increased from 1.7% with UPS to 13.6% with NPS, corresponding to an absolute between-group difference of 11.8 percentage points. Although the relative effect was substantial, spontaneous recall remained modest in absolute terms. This does not negate the value of the intervention, because effective participation in implant safety does not necessarily require patients to memorise every technical identifier. A more realistic objective is that they understand the importance of implant identification, know where the relevant information is stored and recognise whom to contact when a concern or safety alert arises. From this perspective, improved recall, greater awareness of traceability and more frequent contact with the surgical team represent complementary dimensions of patient preparedness.

The clinical relevance of implant-related education therefore lies in the patient's ability to mobilise information when a problem arises rather than in factual recall alone. Patients who understand the need for follow-up, recognise the possibility of delayed adverse events and know how to contact the appropriate clinical team may be better positioned to respond to early symptoms or regulatory safety communications. In the present study, improved vigilance-related knowledge was accompanied by greater use of the direct surgical contact pathway, suggesting that the information provided was translated into at least one observable healthcare-seeking behaviour.

### 4.2 Contribution to previous research

Previous studies of education for implant recipients have predominantly focused on immediate or short-term outcomes, including procedural knowledge, perioperative preparedness, anxiety, expectations, satisfaction and postoperative recommendations.^2^, ^11^, ^12^, ^20^, ^21^, ^22^, ^23^ Educational interventions have generally improved patient knowledge, but their content, intensity, follow-up duration and outcome measures have varied considerably. We identified no published study reporting a directly comparable estimate of spontaneous recall of the patient-specific implant manufacturer, brand or model six months after implantation. Direct numerical comparison with the primary outcome of POSTIMP is therefore not possible. Previous studies provide relevant contextual evidence on implant-related knowledge and information retention, but have generally assessed different knowledge domains, earlier time points, or non-patient-specific outcomes.

In orthopaedic surgery, Billon et al., prospectively assessed patients' knowledge and informational needs before and after hip or knee arthroplasty and identified persistent discrepancies between the information transmitted by surgeons and the information retained by patients.^18^ This observation is consistent with the rationale underlying POSTIMP: information being delivered does not necessarily ensure that it is perceived as important, understood or retained. The present trial extends this work by evaluating a structured educational service and by assessing information retrieval six months after surgery.

Targeted educational interventions have also improved device-related knowledge in other implant contexts. Piredda et al., demonstrated improved knowledge among patients receiving structured information concerning totally implantable venous access ports,^22^ while Jeppson et al., reported improved understanding of sacral nerve stimulation following video-based education.^23^ Joshi et al.,^17^ developed and validated a patient-information booklet for ureteric stents, and Abt et al.,^19^ evaluated the influence of education on symptoms and morbidity associated with indwelling ureteral stents. These studies support the value of structured and device-specific information, although most focused on procedural understanding, immediate knowledge or short-term management.

POSTIMP differs from much of this literature by focusing on patient-specific implant identification, traceability and medical-device vigilance at six months. The primary response was verified against the individual hospital traceability record rather than assessed solely through perceived knowledge, satisfaction or a generic questionnaire. The intervention was also evaluated across both orthopaedic and breast implant pathways, suggesting that some educational principles may be transferable between implant specialties.

Across implant types, patients express broadly similar informational needs concerning the device itself, postoperative recovery, daily life, follow-up and possible complications. However, long-term implant identification, traceability and surveillance remain inconsistently addressed. Mayeranoff et al.,^16^ similarly highlighted the need to strengthen patient information throughout the lifespan of an implant. POSTIMP therefore contributes specifically to the less frequently studied domains of implant identification, long-term traceability and vigilance.

### 4.3 Recall, recognition and access to implant documentation

The distinction between spontaneous recall, list-assisted recognition and access to documentation is clinically relevant. Spontaneous recall reflects the ability to retrieve an implant name without external support, whereas recognition assesses whether a familiar manufacturer, brand or model can be identified when a cue is provided. In practice, regulatory safety communications generally identify the devices concerned. Recognition may therefore represent a relevant ability when patients encounter a device alert.

In POSTIMP, list-assisted recognition alone did not differ significantly between groups, whereas the combined outcome of spontaneous recall or recognition was higher with NPS. Because list-assisted recognition was introduced after recruitment had begun, these results should remain exploratory. Future studies should prespecify and assess spontaneous recall, recognition and documentary retrieval as separate outcomes, since they represent distinct cognitive and practical competencies.

The intervention increased the proportion of participants reporting receipt of an implant card or traceability form, but it did not increase the proportion able to read the exact batch or serial number. This suggests that NPS may have increased awareness of the existence and importance of traceability documentation without clearly improving access to the individual identifier.

Patient memory should therefore not be considered a substitute for reliable documentation. Structured education should help patients understand why traceability documents matter, where they should be stored and how they should be used, rather than transferring responsibility for traceability from healthcare institutions to patients. The low level of spontaneous recall, including after NPS, reinforces the need for durable implant cards, institutional traceability systems and reliable patient access to device records.

Improved awareness of adverse-event self-reporting may also strengthen patients' preparedness for medical-device vigilance. Nevertheless, the study did not expose participants to an actual safety alert and did not assess whether they subsequently submitted an appropriate report or correctly determined whether their implant was affected. The observed effect therefore concerns knowledge of the reporting pathway and readiness to act rather than demonstrated reporting effectiveness.

### 4.4 Educational mechanisms

Both groups received an implant identification document, the same implant-specific booklet, an oral presentation and an opportunity to ask questions. The trial did not therefore compare education with no education or written information with face-to-face counselling. Rather, it evaluated the additional contribution of a longer, structured and interactive consultation incorporating active questioning, immediate corrective feedback, an implant-specific image and systematic discussion of identification, traceability and medical-device vigilance.

Several components may have contributed to the observed effect. The quiz required patients to retrieve and actively process information rather than receive it passively. Immediate feedback allowed inaccurate or incomplete understanding to be corrected during the consultation. Combining oral explanations, a visual representation of the implant and a written booklet may have provided multiple cues for subsequent retrieval. Referring participants to the relevant section of the booklet when correcting their answers may also have strengthened the association between the information discussed orally and the durable written resource available after discharge.

The literature supports multimodal and interactive education but does not establish that one medium is consistently superior. Lukkanasomboon et al.,^30^ reported that multimedia information improved knowledge and supported decision-making in patients considering dental implant surgery. Conversely, Camacho-Alonso et al.,^31^ found that audiovisual information increased anxiety and fear compared with conventional verbal information in another dental implant population. These apparently contrasting findings suggest that effectiveness depends not only on the medium but also on the content, timing, patient characteristics and way in which the information is contextualised.

Li et al., reported benefits from phased written education combined with ongoing two-way communication after heart valve replacement.^32^ Platt et al., proposed a multidisciplinary educational framework for patients considering postmastectomy breast reconstruction.^33^ The value of NPS may therefore lie less in the addition of a specific visual or written medium than in the combination of structured content, active patient participation, corrective feedback and access to reusable materials.

The presence of a between-group difference at six months indicates that some effects of the intervention remained measurable at that time. It does not demonstrate that knowledge remained stable throughout the six-month period or that the effect would persist beyond this assessment. Repeated evaluations would be required to characterise the trajectory and durability of retention.

Because NPS was intentionally designed as a multicomponent intervention, the respective contributions of proactive questioning, the quiz, the visual representation, the additional consultation time, corrective feedback and detailed counselling cannot be separated. Future factorial or process-evaluation studies could help identify which components provide the greatest benefit and which are essential for implementation.

### 4.5 Healthcare-seeking behaviour and postoperative pathway navigation

The higher number of postoperative calls in the NPS group is one of the most clinically informative findings of the study. A patient who contacts the implanting department rather than waiting, consulting an unrelated professional or attending an emergency department may be demonstrating greater awareness of the postoperative pathway and a better understanding of where implant-specific expertise is located. Such contacts may allow the surgical team to distinguish expected postoperative symptoms from warning signs, provide tailored advice and determine whether clinical assessment is required.

This finding can therefore be interpreted as a possible marker of appropriate patient activation rather than simply increased healthcare consumption. The absence of a parallel increase in emergency department attendance or hospitalisation is compatible with this interpretation. Moreover, participants receiving NPS had better knowledge of follow-up and vigilance procedures, which strengthens the coherence between the cognitive and behavioural findings.

No statistically significant overall difference was observed in consultations outside the study hospital, emergency department attendance or hospitalisation. However, subgroup estimates suggested that the relationship between NPS and hospitalisation may differ according to the surgical context. Among participants undergoing primary implantation, the estimate was compatible with fewer implant-related hospitalisations under NPS, whereas the estimate among revision patients was less favourable and substantially more imprecise.

One possible interpretation is that structured education may be more effective in relatively uncomplicated primary implantation pathways, where early symptom recognition and direct contact with the surgical team may prevent avoidable escalation. In revision surgery, hospitalisation risk may be driven more strongly by baseline clinical complexity or established complications, reducing the extent to which an educational intervention can modify subsequent outcomes. This interpretation remains exploratory but provides a clinically coherent hypothesis for future adequately powered studies.

Because the reasons and consequences of postoperative calls were not adjudicated, the proposed mechanism cannot be confirmed. Future studies should document the indication, clinical response and subsequent outcome of these contacts.

POSTIMP was powered for a knowledge outcome rather than uncommon clinical events. Although some educational interventions have reduced postoperative opioid use or surgical cancellations [11,12,34], the healthcare-utilisation findings of POSTIMP should be considered hypothesis-generating rather than evidence of resource reduction.^11^, ^12^, ^34^

High satisfaction and confidence levels in both groups may reflect ceiling effects. UPS already included an individual pharmacist consultation, oral presentation of the booklet and an opportunity to ask questions. NPS therefore improved specific knowledge and behavioural domains without substantially changing overall satisfaction with the pharmaceutical service or general confidence in the implant.

### 4.6 Subgroup findings and individual adaptation

The prespecified subgroup analyses suggest that the intervention may affect different knowledge and behavioural domains according to patient characteristics. Younger participants showed a greater improvement in knowledge of implant materials, whereas participants with lower educational attainment showed a greater improvement in awareness of follow-up requirements. Healthcare professional status was associated with differences in list-assisted recognition, and implant type appeared to influence some technical knowledge outcomes.

These findings may reflect differences in previous knowledge, health literacy, familiarity with technical terminology, perceived relevance or preferred learning strategies. They support an adaptive model in which all patients receive a common core of implant information, while the depth, vocabulary, repetition and practical emphasis of the consultation are adjusted to individual needs.

Patients with lower educational attainment may particularly benefit from structured explanations of follow-up and care pathways, whereas those with greater prior healthcare knowledge may more readily retain technical terminology or recognise implant names from a predefined list. Similarly, older patients may require greater repetition, simplified terminology or more emphasis on practical actions than on technical device composition.

The subgroup findings should not be used to restrict access to NPS. The analyses involved multiple interaction tests, small subgroup sizes and several imprecise estimates. Their principal contribution is to identify dimensions that should be prospectively evaluated when adapting the service. A common educational core with individual tailoring appears more appropriate than limiting the intervention according to age, education, implant type or professional background.

### 4.7 Role of the pharmacist and implications for practice

The intervention illustrates an extension of pharmaceutical care from medicines to implantable healthcare products. Pharmacists involved in implant pathways contribute to technical assessment, procurement, traceability, vigilance and safe use. Their counselling competencies may allow them to translate device-related information into accessible messages, identify misunderstandings and connect implant characteristics with practical follow-up and reporting procedures.

The pharmacist's role in NPS was not limited to providing standardised documents. It involved eliciting existing knowledge, adapting explanations to the patient's questions and pace, providing immediate feedback and linking patient-specific implant information to actions that might be required during long-term follow-up. This interactive contribution is relevant because merely providing a document does not ensure that its content is understood, perceived as important or retained.

The study did not compare pharmacists with surgeons, nurses or other professionals delivering the same intervention. It therefore does not demonstrate that pharmacists are the only or most effective professionals for this role. Rather, it shows that trained hospital pharmacists can integrate a structured implant education service into an existing multidisciplinary postoperative pathway.

From an implementation perspective, NPS required approximately 15 additional minutes compared with UPS, trained pharmacists, access to patient-specific implant data and standardised educational materials. Wider implementation should therefore evaluate pharmacist workload, organisational feasibility, intervention fidelity, patient and professional acceptability and cost-effectiveness.

Digital tools and patient portals may provide opportunities to reinforce information after discharge. However, studies of websites, online information and artificial intelligence consistently report variability in readability, completeness and scientific accuracy. Such resources should therefore complement rather than replace professional counselling. The intervention evaluated in POSTIMP could form the initial face-to-face component of a longer educational pathway incorporating reminders, validated digital resources and continued access to implant documentation.

This model should not be interpreted as replacing education provided by surgeons or nurses. Rather, the respective professional contributions are complementary, and pharmacist-led implant education should be integrated within the multidisciplinary patient pathway. A broader postoperative pharmaceutical consultation may also allow device-related education to be linked with medication-related issues arising from the same surgical episode, such as analgesic review and de-escalation after joint replacement. This integrated approach may optimise the clinical contribution of the pharmacist rather than create a separate device-specific activity. Nevertheless, the additional information volume and consultation time may increase workload, and implementation should therefore be organised within the existing care pathway and evaluated for feasibility.

### 4.8 Strengths and limitations

The principal strengths of POSTIMP include its randomised design, centralised allocation, concealment until completion of the baseline variables and blinded six-month assessment. The primary response was verified against the hospital implant traceability record, providing an objective patient-specific reference. The assessment at six months extended beyond the immediate perioperative period evaluated in most educational studies. The inclusion of orthopaedic and breast implant recipients also allowed the same educational strategy to be evaluated across two distinct surgical specialties.

The intervention was delivered by four pharmacists who had received theoretical, simulation-based and supervised workplace training. This supports the feasibility of implementing the service through a trained pharmacy team rather than relying on a single highly specialised individual. The use of usual pharmaceutical support as an active comparator also indicates that the observed effect resulted from the structured and interactive components added to an already established information pathway.

Several limitations should nevertheless be considered. Both participating surgical centres were located within the same university hospital and shared the same pharmaceutical organisation, which may limit transferability to institutions with different implant pathways or professional roles. The questionnaire was developed specifically for POSTIMP and did not constitute a validated psychometric instrument. Although this approach was appropriate for patient-specific factual and actionable outcomes, it limits direct comparison with other studies.

Baseline implant-related knowledge was not measured; consequently, the study estimates the randomised between-group effect at six months rather than within-participant change in knowledge from baseline. Randomisation and the balance achieved for the prespecified minimisation factors reduce the likelihood of systematic baseline differences between groups, but cannot directly estimate individual knowledge gain. Although participants were advised to contact the surgical department for implant-related questions and were cautioned against relying on unverified online information, adherence to this advice was not assessed, and information obtained from other professionals, relatives, patient organisations, or online resources during follow-up was not recorded. Pharmacists delivering the intervention could not be blinded, and participants may have inferred their allocation from the consultation duration or interactive content. No formal assessment of intervention fidelity was conducted, and adaptation to individual patient questions may have produced variation in delivery. NPS was deliberately designed as a multicomponent educational service in which longer consultation time, proactive questioning, corrective feedback and structured implant-specific content were integral components of the intervention. The trial therefore estimates the effect of the structured service as a whole and cannot determine the independent contribution of educational content, increased interaction or the approximately 15 additional minutes of pharmacist contact.

Several secondary outcomes and healthcare-utilisation variables were self-reported and were not verified against medical records. The reasons and clinical consequences of postoperative calls were not recorded, which limits interpretation of whether these contacts represented early appropriate management, reassurance-seeking or unnecessary use. List-assisted recognition was introduced after recruitment had begun and should be considered exploratory.

The primary effect estimate was based on a small number of correct spontaneous recall events, particularly in the UPS group, resulting in a wide confidence interval (OR 9.02, 95% CI 2.49–57.9). Although the point estimate suggests a large relative effect, the magnitude of this effect is therefore statistically imprecise. The limited sizes of several device-specific and patient subgroups similarly resulted in wide confidence intervals. Penalised regression reduced difficulties associated with sparse events but could not remove the underlying imprecision. Finally, the trial was powered for the primary knowledge outcome rather than for clinical events, healthcare utilisation or subgroup effects.

## 5 Conclusion

Compared with usual pharmaceutical support, structured pharmacist-led implant education improved medium-term retention of patient-specific implant information, knowledge of follow-up and vigilance procedures, and direct postoperative engagement with the surgical team. The combination of better information retention, more frequent use of the appropriate contact pathway and no increase in emergency attendance or hospitalisation suggests that the intervention may support earlier and better-directed responses to postoperative concerns. Although prevention of complications or hospital admissions was not demonstrated, these findings provide a clinically coherent basis for integrating structured implant education into postoperative pharmaceutical care. Patient education should complement, rather than replace, durable implant documentation and institutional traceability systems. Further studies should evaluate repeated reinforcement, verified reasons and consequences of postoperative contacts, implementation in independent institutions and effects on clinical outcomes and healthcare utilisation.

## CRediT authorship contribution statement

**Lionel Tortolano:** Writing – review & editing, Writing – original draft, Visualization, Validation, Supervision, Project administration, Methodology, Investigation, Formal analysis, Data curation, Conceptualization. **Nadia Oubaya:** Validation, Methodology. **Jeremy Hacoon:** Formal analysis. **Muriel Paul:** Visualization. **Thomas Brégaint:** Investigation. **Laura Benichou:** Investigation. **Charles-Henri Flouzat Lachaniette:** Investigation. **Romain Bosc:** Methodology, Investigation, Conceptualization. **Quentin Misandeau:** Project administration, Methodology, Investigation, Conceptualization. **Valérie Archer:** Visualization, Conceptualization.

## Funding

This research did not receive any specific grant from funding agencies in the public, commercial, or not-for-profit sectors.

## Declaration of competing interest

The authors declare that they have no known competing financial interests or personal relationships that could have appeared to influence the work reported in this paper.

## Data Availability

The data supporting the findings of this study are available from the corresponding author upon reasonable request.
