# Supplementary material for: Impact of a pharmacist-led implant education service on patient knowledge and healthcare-seeking behaviour: the POSTIMP randomised controlled trial
Source: Explor Res Clin Soc Pharm. 2026 Sep 4;24:100840. doi: 10.1016/j.rcsop.2026.100840 (PMC13572757; doi:10.1016/j.rcsop.2026.100840)
Supplement: Supplementary file 1 [file mmc2.pdf]

COMITE DE PROTECTION DES PERSONNES EST I

CENTRE HOSPITALIER LA CHARTREUSE
1 BOULEVARD CHANOINE KIR – BP 23314
21033 DIJON CEDEX

EXTRAIT DU PROCÈS VERBAL

RÉUNION DU JEUDI 13 FEVRIER 2020

PERSONNES PRESENTES :

Médecins ou personnes qualifiées en matière de recherche biomédicale *(Catégorie I):*
Dr QUENOT (Titulaire) ; M. WHITE (Titulaire) ; Mme SOUDRY-FAURE (Suppléante)
Médecins généralistes *(Catégorie II)* : Dr MAIZIERES (Suppléant)
Pharmaciens Hospitalier *(Catégorie III)* : M. BOULIN (Titulaire)
Infirmiers et Infirmières *(Catégorie IV)* : Mme PERROT (Suppléante)
Personnes qualifiées en raison de leur compétence à l'égard des questions d'éthique *(Catégorie V)*:
Mme POURREZ (Titulaire)
Psychologue *(Catégorie VI)* : Mme BALAS (Titulaire)
Travailleur social *(Catégorie VII)* : Mme GONNEAUD (Titulaire)
Personnes qualifiées en raison de leur compétence en matière juridique (*Catégorie VIII)*:
Mme TABUTIAUX (Titulaire)
Représentants des associations agréées de malades et d'usagers du système de santé *(Catégorie IX):*
M. LECOMTE (Titulaire) ; Mme PLASSARD (Titulaire)

Au cours de cette réunion a été étudié le dossier :

N° SI 20.01.20.50011 – IDRCB 2019-A03187-50
«Étude randomisée, contrôlée, de supériorité entre 2 types d'accompagnements pharmaceutiques postopératoires sur la connaissance des patients de leur d'IMPlant : prothèses mammaires ou articulaires (prothèses de hanche ou de genou), à 6 mois POSTopératoire »
Promoteur : **ASSISTANCE PUBLIQUE – HÔPITAUX DE PARIS**
Investigateur principal: **Dr TORTOLANO**

Avis du Comité : FAVORABLE

Documents examinés par le Comité :

- *Courrier de demande d'avis au CPP – signé et daté du 13/01/2020*
- *Formulaire de demande d'autorisation auprès de l'Agence Nationale de Sécurité du Médicament et des produits de santé ou de demande d'avis au Comité de Protection des Personnes pour une recherche mentionnée au 1° ou au 2° de l'article L.1121-1 du Code de la Santé Publique ne portant pas sur un produit mentionné à l'article L.5311-1 du Code de la Santé Publique – signé et daté du 22/01/2020*
- *Document additionnel à la demande d'avis au Comité de Protection des Personnes sur un projet de recherche mentionnée au 1° ou au 2° de l'article L.1121-1 du code de la santé publique ne portant pas sur un produit mentionné à l'article L.5311-1 du même code – signé et daté du 13/01/2020*
- *Protocole POSTIMP – version 1.0 du 13/12/2019 et page de signature*
- *Résumé du protocole POSTIMP – version 1.0 du 13/12/2019*
- *Description du contenu des accompagnements pharmaceutiques à l'attention des membres du comité de protection des personnes – version 1.0 du 23/01/2020*
- *Fiche Prothèses mammaires et hypoplasie des seins ou Plastie d'augmentation mammaire par prothèses – version 9 de janvier 2019*
- *Fiche Reconstruction du sein par prothèse – version 7 de janvier 2019*
- *Livret de conseils pratiques « Vivre avec votre prothèse de genou » - version du 13/04/2018*

…/…

- *Livret de conseils pratique « Vivre avec votre prothèse de hanche mise en place par voie antérieure » - version avril 2018*
- *Livret de conseils pratique « Vivre avec votre prothèse de hanche mise en place par voie postéro-externe » - version avril 2018*
- *Quizz APN Prothèse de genou – version 1.0 du 20/01/2020*
- *Quizz APN Prothèse de hanche posée par voie antérieure – version 1.0 du 20/01/2020*
- *Quizz APN Prothèse de hanche posée par voie postéro-externe – version 1.0 du 20/01/2020*
- *Quizz APN Prothèse mammaire – version 2.0 du 20/01/2020*
- *Justification de l'adéquation des moyens – signée et datée du 13/01/2020*
- *Attestation d'assurance BioMedicinsure n° 0100518814033 200009 – signée et datée du 10/01/2020*
- *Lettre d'information et Formulaire de consentement Patient majeur – version 1.0 du 13/12/2019*
- *CV du Dr Charles-Henri FLOUZAT-LACHANIETTE*
- *CV du Dr Lionel TORTOLANO*
- *CV du Dr Romain BOSC*
- *Attestation aux BPC du Dr Lionel TORTOLANO – datée du 21/12/2019*

Fait à Dijon, le 18 février 2020

Le Président
Dr Jean-Pierre QUENOT
