# Supplementary material for: Impact of a pharmacist-led implant education service on patient knowledge and healthcare-seeking behaviour: the POSTIMP randomised controlled trial
Source: Explor Res Clin Soc Pharm. 2026 Sep 4;24:100840. doi: 10.1016/j.rcsop.2026.100840 (PMC13572757; doi:10.1016/j.rcsop.2026.100840)
Supplement: Supplementary file 2 [file mmc1.docx]

Supplementary data

**Table S1: Table one according to availability of the primary endpoint - %, given per row**

| Characteristic | Available, N = 207^1^ | Missing, N = 28^1^ | Overall, N = 235^1^ |
| --- | --- | --- | --- |
| Arm |  |  |  |
| UPS | 100 (85.5%) | 17 (14.5%) | 117 (100.0%) |
| NPS | 107 (90.7%) | 11 (9.3%) | 118 (100.0%) |
| Center |  |  |  |
| Orthopaedic surgery | 172 (87.8%) | 24 (12.2%) | 196 (100.0%) |
| Plastic surgery | 35 (89.7%) | 4 (10.3%) | 39 (100.0%) |
| Sex |  |  |  |
| Male | 80 (84.2%) | 15 (15.8%) | 95 (100.0%) |
| Female | 127 (90.7%) | 13 (9.3%) | 140 (100.0%) |
| Age | 60.0 (16.6) | 52.3 (19.6) | 59.0 (17.1) |
| Level of study |  |  |  |
| Elementary school/study certificate | 50 (87.7%) | 7 (12.3%) | 57 (100.0%) |
| Middle school diploma | 38 (82.6%) | 8 (17.4%) | 46 (100.0%) |
| High school diploma | 32 (91.4%) | 3 (8.6%) | 35 (100.0%) |
| Post-High school diploma | 87 (89.7%) | 10 (10.3%) | 97 (100.0%) |
| French speaking | 206 (88.0%) | 28 (12.0%) | 234 (100.0%) |
| Experience as a health care professional | 33 (97.1%) | 1 (2.9%) | 34 (100.0%) |
| Prosthesis type |  |  |  |
| THR | 105 (87.5%) | 15 (12.5%) | 120 (100.0%) |
| TKR | 67 (88.2%) | 9 (11.8%) | 76 (100.0%) |
| Breast implant | 35 (89.7%) | 4 (10.3%) | 39 (100.0%) |
| Primo-implantation/reprise |  |  |  |
| Primary implantation | 179 (87.3%) | 26 (12.7%) | 205 (100.0%) |
| Resumption | 28 (93.3%) | 2 (6.7%) | 30 (100.0%) |
| Brand / manufacturer of the breast implant |  |  |  |
| Sebbin | 31 (88.6%) | 4 (11.4%) | 35 (100.0%) |
| Motiva | 4 (100.0%) | 0 (0.0%) | 4 (100.0%) |
| Unknown | 172 | 24 | 196 |
| Brand / manufacturer of the prosthesis (THR/TKR) |  |  |  |
| Medacta | 66 (86.8%) | 10 (13.2%) | 76 (100.0%) |
| Osteal medical (=ceraver) | 69 (89.6%) | 8 (10.4%) | 77 (100.0%) |
| Groupe Lepine | 17 (89.5%) | 2 (10.5%) | 19 (100.0%) |
| SYMBIOS | 18 (85.7%) | 3 (14.3%) | 21 (100.0%) |
| Depuy Synthes (Johnson & Johnson) | 1 (100.0%) | 0 (0.0%) | 1 (100.0%) |
| Medicalex | 1 (100.0%) | 0 (0.0%) | 1 (100.0%) |
| STANMORE STRYKER | 0 (0.0%) | 1 (100.0%) | 1 (100.0%) |
| Unknown | 35 | 4 | 39 |
| Indication (pour les prothèses mammaires) |  |  |  |
| Breast augmentation surgery | 20 (87.0%) | 3 (13.0%) | 23 (100.0%) |
| Breast reconstruction surgery | 15 (93.8%) | 1 (6.3%) | 16 (100.0%) |
| Unknown | 172 | 24 | 196 |
| Indication for the placement of the prosthesis (THR) |  |  |  |
| Osteoarthritis | 67 (93.1%) | 5 (6.9%) | 72 (100.0%) |
| Aseptic osteonecrosis | 30 (76.9%) | 9 (23.1%) | 39 (100.0%) |
| Dysplasia | 4 (100.0%) | 0 (0.0%) | 4 (100.0%) |
| Other | 4 (80.0%) | 1 (20.0%) | 5 (100.0%) |
| Unknown | 102 | 13 | 115 |
| Indication for the placement of the prosthesis (TKR) |  |  |  |
| Osteoarthritis | 64 (88.9%) | 8 (11.1%) | 72 (100.0%) |
| Other | 3 (75.0%) | 1 (25.0%) | 4 (100.0%) |
| Unknown | 140 | 19 | 159 |
| ^1^n (%, given per row); Mean (SD) | | | |

**Table S2: Primary and secondary outcomes in the per protocol population**

| Characteristic | N | UPS, N = 100^1^ | NPS, N = 106^1^ | OR | CI | p-value |
| --- | --- | --- | --- | --- | --- | --- |
| Correct answer: manufacturer/brand or model (from memory) | 206 | 1 (1.0%) N=100 | 14 (13.2%) N=106 | 15.1 | 2.94-276 | **0.009** |
| Correct answer: manufacturer/brand or model (with the help of a list among those who failed from memory)^2^ | 110 | 10 (16.7%) N=60 | 7 (14.0%) N=50 | 0.810 | 0.270-2.30 | 0.700 |
| Correct answer: manufacturer/brand or model (from memory or with the help of a list)^2^ | 125 | 11 (18.0%) N=61 | 21 (32.8%) N=64 | 2.22 | 0.980-5.27 | 0.061 |
| Correct answer: establishment of the prosthesis placement | 206 | 96 (96.0%) N=100 | 103 (97.2%) N=106 | 1.43 | 0.310-7.42 | 0.645 |
| Correct answer: regular medical follow-up required? | 204 | 61 (62.2%) N=98 | 82 (77.4%) N=106 | 2.07 | 1.13-3.86 | **0.019** |
| Correct answer: prosthesis materials | 206 | 26 (26.0%) N=100 | 67 (63.2%) N=106 | 4.89 | 2.72-9.00 | **<0.001** |
| Correct answer: did you receive the patient-implant card (BI only) | 35 | 16 (100.0%) N=16 | 18 (94.7%) N=19 | 0.370 | 0.000-7.51 |  |
| Correct answer: did you receive the patient-implant form (THR/TKR only) | 171 | 57 (67.9%) N=84 | 74 (85.1%) N=87 | 2.70 | 1.30-5.84 | **0.009** |
| Correct answer: did you receive the patient-implant card or form (pooled) | 206 | 73 (73.0%) N=100 | 92 (86.8%) N=106 | 2.43 | 1.21-5.08 | **0.015** |
| Correct answer: date of prosthesis placement | 206 | 81 (81.0%) N=100 | 88 (83.0%) N=106 | 1.15 | 0.560-2.35 | 0.706 |
| Correct answer: surgeon's name | 206 | 87 (87.0%) N=100 | 89 (84.0%) N=106 | 0.780 | 0.350-1.70 | 0.537 |
| Correct answer: prosthesis batch number (THR/TKR) | 171 | 42 (50.0%) N=84 | 45 (51.7%) N=87 | 1.07 | 0.590-1.96 | 0.822 |
| Correct answer: breast implant serial number | 35 | 12 (75.0%) N=16 | 14 (73.7%) N=19 | 0.930 | 0.190-4.33 | 0.929 |
| Correct answer: batch or serial number (THR/TKR or BI) | 206 | 54 (54.0%) N=100 | 59 (55.7%) N=106 | 1.07 | 0.620-1.85 | 0.811 |
| Correct answer to the 3 items: date of prosthesis placement, surgeon's name, batch or serial number | 206 | 46 (46.0%) N=100 | 52 (49.1%) N=106 | 1.13 | 0.650-1.96 | 0.661 |
| Correct answer: texturing of your prosthesis surface (BI only) | 35 | 4 (25.0%) N=16 | 13 (68.4%) N=19 | 6.50 | 1.57-32.1 | **0.014** |
| Correct answer: scar at the front or at the back of the pelvis? (THR only) | 104 | 47 (90.4%) N=52 | 52 (100.0%) N=52 | 12.2 | 1.32-1,612 |  |
| Correct answer: fixed prosthesis with or without cement? (THR only) | 104 | 19 (36.5%) N=52 | 26 (50.0%) N=52 | 1.74 | 0.800-3.84 | 0.167 |
| Knowledge of the possibility of self-reporting an adverse event associated with the prosthesis | 206 | 58 (58.0%) N=100 | 81 (76.4%) N=106 | 2.35 | 1.30-4.31 | **0.005** |
| Correct answer: Who can you report a problem with your prosthesis to? | 206 |  |  | 1.00 | NA-NA | >0.999 |
| yes |  | 100 (100.0%) N=100 | 106 (100.0%) N=106 |  |  |  |
| Level of confidence in the prosthesis (categorical from Lickert scale) | 206 |  |  | 1.25 | 0.580-2.75 | 0.571 |
| Neutral to not confident at all |  | 16 (16.0%) N=100 | 14 (13.2%) N=106 |  |  |  |
| Totally or somewhat confident |  | 84 (84.0%) N=100 | 92 (86.8%) N=106 |  |  |  |
| Number of calls to the care unit where the patient was hospitalized after the prosthesis was placed | 206 |  |  | 2.84 | 1.24-7.14 | **0.018** |
| 0 |  | 92 (92.0%) N=100 | 85 (80.2%) N=106 |  |  |  |
| At least 1 |  | 8 (8.0%) N=100 | 21 (19.8%) N=106 |  |  |  |
| Number of consultations related to the prosthesis with a surgeon outside the hospital Henri Mondor | 206 |  |  | 0.390 | 0.080-1.44 | 0.178 |
| 0 |  | 93 (93.0%) N=100 | 103 (97.2%) N=106 |  |  |  |
| At least 1 |  | 7 (7.0%) N=100 | 3 (2.8%) N=106 |  |  |  |
| Admission to the emergency room for a complication related to the prosthesis | 206 | 8 (8.0%) N=100 | 8 (7.5%) N=106 | 0.940 | 0.330-2.65 | 0.903 |
| Hospitalization for a complication related to the prosthesis | 205 | 11 (11.1%) N=99 | 11 (10.4%) N=106 | 0.930 | 0.380-2.27 | 0.865 |
| Satisfaction with the information provided about the prosthesis after the operation (categorical from Lickert scale) | 180 |  |  | 1.25 | 0.530-2.94 | 0.607 |
| Quite to not satisfed |  | 13 (15.3%) N=85 | 12 (12.6%) N=95 |  |  |  |
| Satisfied |  | 72 (84.7%) N=85 | 83 (87.4%) N=95 |  |  |  |
| Do you need information about your implant or your convalescence ?^2^ | 63 | 30 (85.7%) N=35 | 23 (82.1%) N=28 | 0.770 | 0.190-3.06 | 0.700 |
| ^1^n (%) N=N | | | | | | |
| ^2^Collected for a part only because variable added during the study <br>Asked for the 'manufacturer/brand or model' item for n = 60 in UPS Arm, and n = 50 in NPS Arm, before imputation. <br>Asked for 'need information' item for n = 35 in UPS Arm, and n = 28 in NPS Arm, in the per protocol population | | | | | | |

**Table S3:primary and secondary outcomes in the intention-to-treat population: univariate analyses and analyses adjusting for minimisation criteria**

| **Characteristic** | **N** | **UPS, N = 117^1^** | **NPS, N = 118^1^** | **Crude  OR** | **95% CI** | **p-value** | **Adjusted OR** | **95%CI** | **p-value** |
| --- | --- | --- | --- | --- | --- | --- | --- | --- | --- |
| Correct answer: manufacturer/brand or model (from memory) | 235 | 2 (1.7%) N=117 | 16 (13.6%) N=118 | 9.02 | 2.49-57.9 | **0.004** | 8.84 | 2.48, 48.4 | **<0.001** |
| Correct answer: manufacturer/brand or model (with the help of a list among those who failed from memory)^2^ | 217 | 13 (11.3%) N=115 | 19 (18.6%) N=102 | 1.80 | 0.840-3.93 | 0.13 | 2.68 | 1.10, 6.97 | **0.035** |
| Correct answer: manufacturer/brand or model (from memory or with the help of a list)^2^ | 235 | 15 (12.8%) N=117 | 35 (29.7%) N=118 | 2.87 | 1.49-5.75 | **0.002** | 3.96 | 1.82, 9.24 | **<0.001** |
| Correct answer: establishment of the prosthesis placement | 235 | 113 (96.6%) N=117 | 115 (97.5%) N=118 | 1.36 | 0.290-7.02 | 0.69 | 0.99 | 0.19, 4.94 | 0.9 |
| Correct answer: regular medical follow-up required? | 235 | 68 (58.1%) N=117 | 94 (79.7%) N=118 | 2.82 | 1.59-5.10 | **<0.001** | 2.97 | 1.63, 5.51 | **<0.001** |
| Correct answer: prosthesis materials | 235 | 29 (24.8%) N=117 | 79 (66.9%) N=118 | 6.15 | 3.52-11.0 | **<0.001** | 7.48 | 4.09, 14.2 | **<0.001** |
| Correct answer: did you receive the patient-implant card (BI only) | 39 | 19 (100.0%) N=19 | 19 (95.0%) N=20 | 0.330 | 0.000-6.65 |  | 0.36 | 0.01, 6.13 | 0.5 |
| Correct answer: did you receive the patient-implant form (THR/TKR only) | 196 | 69 (70.4%) N=98 | 85 (86.7%) N=98 | 2.75 | 1.35-5.85 | **0.006** | 2.95 | 1.38, 6.62 | **0.007** |
| Correct answer: did you receive the patient-implant card or form (pooled) | 235 | 88 (75.2%) N=117 | 104 (88.1%) N=118 | 2.45 | 1.24-5.04 | **0.012** | 2.62 | 1.25, 5.73 | **0.013** |
| Correct answer: date of prosthesis placement | 235 | 96 (82.1%) N=117 | 100 (84.7%) N=118 | 1.22 | 0.610-2.44 | 0.58 | 1.18 | 0.58, 2.42 | 0.7 |
| Correct answer: surgeon's name | 235 | 103 (88.0%) N=117 | 101 (85.6%) N=118 | 0.810 | 0.370-1.72 | 0.58 | 0.75 | 0.33, 1.66 | 0.5 |
| Correct answer: prosthesis batch number (THR/TKR) | 196 | 54 (55.1%) N=98 | 56 (57.1%) N=98 | 1.09 | 0.620-1.91 | 0.77 | 1.06 | 0.58, 1.95 | 0.8 |
| Correct answer: breast implant serial number | 39 | 15 (78.9%) N=19 | 15 (75.0%) N=20 | 0.800 | 0.170-3.60 | 0.77 | 0.96 | 0.18, 5.25 | 0.9 |
| Correct answer: batch or serial number (THR/TKR or BI) | 235 | 69 (59.0%) N=117 | 71 (60.2%) N=118 | 1.05 | 0.620-1.77 | 0.85 | 1.02 | 0.58, 1.78 | 0.9 |
| Correct answer to the 3 items: date of prosthesis placement, surgeon's name, batch or serial number | 235 | 61 (52.1%) N=117 | 64 (54.2%) N=118 | 1.09 | 0.650-1.82 | 0.75 | 1.07 | 0.62, 1.82 | 0.8 |
| Correct answer: texturing of your prosthesis surface (BI only) | 39 | 6 (31.6%) N=19 | 14 (70.0%) N=20 | 5.06 | 1.36-21.2 | **0.020** | 7.34 | 1.71, 43.8 | **0.006** |
| Correct answer: scar at the front or at the back of the pelvis? (THR only) | 120 | 55 (91.7%) N=60 | 60 (100.0%) N=60 | 12.0 | 1.31-1,588 |  | 9.16 | 0.89, 1,233 | 0.065 |
| Correct answer: fixed prosthesis with or without cement? (THR only) | 120 | 23 (38.3%) N=60 | 33 (55.0%) N=60 | 1.97 | 0.950-4.11 | 0.069 | 2.00 | 0.90, 4.54 | 0.091 |
| Knowledge of the possibility of self-reporting an adverse event associated with the prosthesis | 235 | 69 (59.0%) N=117 | 92 (78.0%) N=118 | 2.46 | 1.40-4.40 | **0.002** | 2.55 | 1.42, 4.67 | **0.002** |
| Correct answer: Who can you report a problem with your prosthesis to? | 235 |  |  | 1.00 | NA-NA | >0.99 | 1.00 | NA-NA | >0.99 |
| yes |  | 117 (100.0%) N=117 | 118 (100.0%) N=118 |  |  |  |  |  |  |
| Level of confidence in the prosthesis (categorical from Lickert scale) | 235 |  |  | 1.18 | 0.550-2.57 | 0.68 | 1.20 | 0.53, 2.75 | 0.7 |
| Neutral to not confident at all |  | 16 (13.7%) N=117 | 14 (11.9%) N=118 |  |  |  |  |  |  |
| Totally or somewhat confident |  | 101 (86.3%) N=117 | 104 (88.1%) N=118 |  |  |  |  |  |  |
| Number of calls to the care unit where the patient was hospitalized after the prosthesis was placed | 235 |  |  | 3.12 | 1.38-7.77 | **0.009** | 3.28 | 1.41, 8.36 | **0.008** |
| 0 |  | 109 (93.2%) N=117 | 96 (81.4%) N=118 |  |  |  |  |  |  |
| At least 1 |  | 8 (6.8%) N=117 | 22 (18.6%) N=118 |  |  |  |  |  |  |
| Number of consultations related to the prosthesis | 235 |  |  | 0.410 | 0.090-1.51 | 0.20 | 0.47 | 0.11, 1.59 | 0.2 |
| 0 |  | 110 (94.0%) N=117 | 115 (97.5%) N=118 |  |  |  |  |  |  |
| At least 1 |  | 7 (6.0%) N=117 | 3 (2.5%) N=118 |  |  |  |  |  |  |
| Admission to the emergency room for a complication related to the prosthesis | 235 | 8 (6.8%) N=117 | 8 (6.8%) N=118 | 0.990 | 0.350-2.78 | 0.99 | 1.63 | 0.49, 6.08 | 0.4 |
| Hospitalization for a complication related to the prosthesis | 235 | 11 (9.4%) N=117 | 11 (9.3%) N=118 | 0.990 | 0.410-2.41 | 0.98 | 0.96 | 0.38, 2.43 | 0.9 |
| Satisfaction with the information provided about the prosthesis after the operation (categorical from Lickert scale) | 235 |  |  | 1.37 | 0.640-3.02 | 0.42 | 1.36 | 0.61, 3.10 | 0.5 |
| Quite to not satisfed |  | 17 (14.5%) N=117 | 13 (11.0%) N=118 |  |  |  |  |  |  |
| Satisfied |  | 100 (85.5%) N=117 | 105 (89.0%) N=118 |  |  |  |  |  |  |
| Do you need information about your implant or your convalescence ?^2^ | 235 | 107 (91.5%) N=117 | 97 (82.2%) N=118 | 0.430 | 0.190-0.940 | **0.040** | 0.29 | 0.09, 0.85 | **0.030** |

**Table S4: Primary and secondary outcomes in per protocol population: univariate analyses and analyses adjusting for minimisation criteria**

| Characteristic | N | estimate | ci | p.value | firth_adj_reg (1=YES ; 0= no) |
| --- | --- | --- | --- | --- | --- |
| Correct answer: manufacturer/brand or model (from memory) | 206 | 10.12 | 2.37, 94.7 | **0.001** | 1 |
| Correct answer: manufacturer/brand or model (with the help of a list among those who failed from memory)^2^ | 110 | 0.63 | 0.18, 2.01 | 0.441 | 1 |
| Correct answer: manufacturer/brand or model (from memory or with the help of a list)^2^ | 125 | 2.01 | 0.84, 5.08 | 0.119 | 1 |
| Correct answer: establishment of the prosthesis placement | 206 | 1.15 | 0.22, 5.89 | 0.86 | 1 |
| Correct answer: regular medical follow-up required? | 204 | 2.05 | 1.09, 3.92 | **0.027** | 0 |
| Correct answer: prosthesis materials | 206 | 5.89 | 3.11, 11.6 | **<0.001** | 0 |
| Correct answer: did you receive the patient-implant card (BI only) | 35 | 0.28 | 0.00, 6.08 | 0.428 | 1 |
| Correct answer: did you receive the patient-implant form (THR/TKR only) | 171 | 2.82 | 1.29, 6.44 | **0.011** | 0 |
| Correct answer: did you receive the patient-implant card or form (pooled) | 206 | 2.51 | 1.17, 5.57 | **0.02** | 0 |
| Correct answer: date of prosthesis placement | 206 | 1.11 | 0.53, 2.33 | 0.781 | 0 |
| Correct answer: surgeon's name | 206 | 0.69 | 0.30, 1.58 | 0.391 | 0 |
| Correct answer: prosthesis batch number (THR/TKR) | 171 | 0.97 | 0.50, 1.85 | 0.916 | 0 |
| Correct answer: breast implant serial number | 35 | 1.06 | 0.18, 6.56 | 0.946 | 1 |
| Correct answer: batch or serial number (THR/TKR or BI) | 206 | 0.99 | 0.54, 1.80 | 0.967 | 0 |
| Correct answer to the 3 items: date of prosthesis placement, surgeon's name, batch or serial number | 206 | 1.09 | 0.61, 1.94 | 0.777 | 0 |
| Correct answer: texturing of your prosthesis surface (BI only) | 35 | 7.46 | 1.58, 51.9 | **0.01** | 1 |
| Correct answer: scar at the front or at the back of the pelvis? (THR only) | 104 | 8.01 | 0.76, 1,092 | 0.09 | 1 |
| Correct answer: fixed prosthesis with or without cement? (THR only) | 104 | 1.72 | 0.73, 4.12 | 0.218 | 0 |
| Knowledge of the possibility of self-reporting an adverse event associated with the prosthesis | 206 | 2.39 | 1.30, 4.48 | **0.006** | 0 |
| Correct answer: Who can you report a problem with your prosthesis to? | 206 | 1.18 | 0.01, 125 | 0.919 | 1 |
| yes |  | NA |  |  |  |
| Level of confidence in the prosthesis (categorical from Lickert scale) | 206 | 1.27 | 0.55, 2.96 | 0.577 | 0 |
| Neutral to not confident at all |  | NA |  |  |  |
| Totally or somewhat confident |  | NA |  |  |  |
| Number of calls to the care unit where the patient was hospitalized after the prosthesis was placed | 206 | 2.96 | 1.24, 7.75 | **0.019** | 0 |
| 0 |  | NA |  |  |  |
| At least 1 |  | NA |  |  |  |
| Number of consultations related to the prosthesis with a surgeon outside the hospital Henri Mondor | 206 | 0.47 | 0.11, 1.59 | 0.228 | 1 |
| 0 |  | NA |  |  |  |
| At least 1 |  | NA |  |  |  |
| Admission to the emergency room for a complication related to the prosthesis | 206 | 1.63 | 0.48, 6.16 | 0.447 | 0 |
| Hospitalization for a complication related to the prosthesis | 205 | 0.90 | 0.36, 2.29 | 0.829 | 0 |
| Satisfaction with the information provided about the prosthesis after the operation (categorical from Lickert scale) | 180 | 1.20 | 0.50, 2.93 | 0.678 | 0 |
| Quite to not satisfed |  | NA |  |  |  |
| Satisfied |  | NA |  |  |  |
| Do you need information about your implant or your convalescence ?^2^ | 63 | 1.03 | 0.22, 5.27 | 0.972 | 0 |

**Table S5: Type of Prosthesis (Breast implant vs. THR/TKR)**

|  | **Breast implant** | | **THR/TKR** | |  |
| --- | --- | --- | --- | --- | --- |
|  | **Odds Ratio (95% CI)** | **P** | **Odds Ratio (95% CI)** | **P** | **P for interaction** |
| Correct answer: manufacturer/brand or model (from memory) | 12.0 (1.86, 238) | **0.027** | 8.62 (1.54, 162) | **0.044** | 0.8 |
| Correct answer: manufacturer/brand or model (with the help of a list among those who failed from memory) | 1.75 (0.40, 8.05) | 0.5 | 2.83 (1.00, 9.22) | 0.060 | 0.6 |
| Correct answer: manufacturer/brand or model (from memory or with the help of a list) | 3.33 (0.89, 13.8) | 0.082 | 3.93 (1.59, 11.2) | **0.005** | 0.8 |
| Correct answer: establishment of the prosthesis placement | 1.05 (0.01, 199) | 0.9 | 1.30 (0.31, 5.95) | 0.7 | 0.9 |
| Correct answer: regular medical follow-up required? | 1.51 (0.29, 8.73) | 0.6 | 3.11 (1.68, 5.90) | **<0.001** | 0.4 |
| Correct answer: prosthesis materials | 41.2 (6.31, 832) | **0.001** | 5.15 (2.81, 9.71) | **<0.001** | 0.078 |
| Correct answer: did you receive the patient-implant card or form (pooled) | 0.33 (0.00, 6.65) | 0.5 | 2.69 (1.33, 5.65) | **0.005** | 0.2 |
| Correct answer: date of prosthesis placement | 0.47 (0.06, 2.76) | 0.4 | 1.44 (0.68, 3.12) | 0.3 | 0.3 |
| Correct answer: surgeon’s name | 0.47 (0.06, 2.76) | 0.4 | 0.91 (0.39, 2.12) | 0.8 | 0.5 |
| Correct answer: batch or serial number (THR/TKR or BI) | 0.80 (0.17, 3.60) | 0.8 | 1.09 (0.62, 1.91) | 0.8 | 0.7 |
| Correct answer to the 3 items: date of prosthesis placement, surgeon’s name, batch or serial number | 0.69 (0.18, 2.58) | 0.6 | 1.18 (0.67, 2.07) | 0.6 | 0.5 |
| Knowledge of the possibility of self-reporting an adverse event associated with the prosthesis | 2.40 (0.41, 19.1) | 0.3 | 2.51 (1.38, 4.67) | **0.003** | 0.9 |
| Correct answer: Who can you report a problem with your prosthesis to? | 1.00 (0.00, Inf) | 0.9 | 1.00 (0.00, Inf) | 0.9 | 0.9 |
| Level of confidence in the prosthesis (categorical from Lickert scale) | 2.24 (0.20, 50.7) | 0.5 | 1.09 (0.48, 2.48) | 0.8 | 0.6 |
| Call to the care unit where the patient was hospitalized after the prosthesis was placed | 14.7 (2.31, 291) | **0.016** | 1.99 (0.78, 5.51) | 0.2 | 0.10 |
| Consultation related to the prosthesis with a surgeon outside of the surgery centre | 0.30 (0.00, 6.00) | 0.4 | 0.52 (0.12, 1.90) | 0.3 | 0.8 |
| Admission to the emergency room for a complication related to the prosthesis | 0.30 (0.00, 6.00) | 0.4 | 1.15 (0.41, 3.28) | 0.8 | 0.4 |
| Hospitalization for a complication related to the prosthesis | 0.17 (0.00, 2.28) | 0.2 | 1.24 (0.50, 3.14) | 0.6 | 0.2 |
| Satisfaction with the information provided about the prosthesis after the operation (categorical from Lickert scale) | 1.06 (0.12, 9.65) | 0.9 | 1.43 (0.62, 3.36) | 0.4 | 0.8 |
| Do you need information about your implant or your convalescence? | 0.15 (0.03, 0.59) | **0.009** | 0.65 (0.16, 2.36) | 0.5 | 0.14 |

**Table S6: Age**

|  | **<65 yo** | | **>=65 yo** | |  |
| --- | --- | --- | --- | --- | --- |
|  | **Odds Ratio (95% CI)** | **P** | **Odds Ratio (95% CI)** | **P** | **P for interaction** |
| Correct answer: manufacturer/brand or model (from memory) | 12.5 (2.33, 233) | **0.017** | 5.56 (0.85, 109) | 0.12 | 0.6 |
| Correct answer: manufacturer/brand or model (with the help of a list among those who failed from memory) | 2.04 (0.89, 4.82) | 0.10 | 1.11 (0.04, 28.7) | 0.9 | 0.7 |
| Correct answer: manufacturer/brand or model (from memory or with the help of a list) | 3.03 (1.42, 6.74) | **0.005** | 3.34 (0.73, 23.6) | 0.2 | 0.9 |
| Correct answer: establishment of the prosthesis placement | 1.03 (0.04, 26.4) | 0.9 | 1.50 (0.24, 11.8) | 0.7 | 0.8 |
| Correct answer: regular medical follow-up required? | 1.86 (0.88, 3.99) | 0.11 | 5.05 (2.05, 13.5) | **<0.001** | 0.10 |
| Correct answer: prosthesis materials | 13.1 (5.93, 31.0) | **<0.001** | 2.60 (1.16, 6.00) | **0.022** | **0.006** |
| Correct answer: did you receive the patient-implant form (THR/TKR only) | 1.66 (0.58, 4.99) | 0.3 | 4.16 (1.56, 12.5) | **0.007** | 0.2 |
| Correct answer: did you receive the patient-implant card or form (pooled) | 1.34 (0.49, 3.74) | 0.6 | 4.35 (1.64, 13.1) | **0.005** | 0.11 |
| Correct answer: date of prosthesis placement | 0.78 (0.26, 2.22) | 0.6 | 1.72 (0.68, 4.58) | 0.3 | 0.3 |
| Correct answer: surgeon’s name | 0.75 (0.23, 2.29) | 0.6 | 0.85 (0.29, 2.42) | 0.8 | 0.9 |
| Correct answer: prosthesis batch number (THR/TKR) | 0.93 (0.41, 2.11) | 0.9 | 1.23 (0.56, 2.72) | 0.6 | 0.6 |
| Correct answer: batch or serial number (THR/TKR or BI) | 0.86 (0.42, 1.75) | 0.7 | 1.32 (0.61, 2.91) | 0.5 | 0.4 |
| Correct answer to the 3 items: date of prosthesis placement, surgeon’s name, batch or serial number | 0.93 (0.47, 1.85) | 0.8 | 1.32 (0.60, 2.90) | 0.5 | 0.5 |
| Correct answer: texturing of your prosthesis surface (BI only) | 5.63 (1.45, 25.2) | **0.017** | 5.63 (1.45, 25.2) | **0.017** | - |
| Correct answer: scar at the front or at the back of the pelvis? (THR only) | 10.4 (1.05, 1,396) | **0.045** | 3.00 (0.15, 447) | 0.5 | 0.6 |
| Correct answer: fixed prosthesis with or without cement? (THR only) | 2.58 (1.02, 6.80) | **0.049** | 1.29 (0.39, 4.32) | **0.7** | 0.4 |
| Knowledge of the possibility of self-reporting an adverse event associated with the prosthesis | 3.31 (1.47, 7.95) | **0.005** | 1.89 (0.85, 4.30) | 0.12 | 0.3 |
| Correct answer: Who can you report a problem with your prosthesis to? | 1.00 (0.00, Inf) | 0.9 | 1.00 (0.00, Inf) | 0.9 | 0.9 |
| Level of confidence in the prosthesis (categorical from Lickert scale) | 1.46 (0.57, 3.83) | 0.4 | 0.77 (0.18, 3.07) | 0.7 | 0.4 |
| Call to the care unit where the patient was hospitalized after the prosthesis was placed | 3.75 (1.37, 12.1) | **0.015** | 2.18 (0.54, 10.8) | 0.3 | 0.6 |
| Consultation related to the prosthesis with a surgeon outside of the surgery centre | 0.08 (0.00, 0.74) | **0.022** | 1.46 (0.27, 9.10) | 0.7 | **0.046** |
| Admission to the emergency room for a complication related to the prosthesis | 0.72 (0.14, 3.37) | 0.7 | 1.31 (0.33, 5.57) | 0.7 | 0.6 |
| Hospitalization for a complication related to the prosthesis | 0.67 (0.19, 2.21) | 0.5 | 1.60 (0.43, 6.62) | 0.5 | 0.3 |
| Satisfaction with the information provided about the prosthesis after the operation (categorical from Lickert scale) | 0.89 (0.29, 2.63) | 0.8 | 2.20 (0.72, 7.54) | 0.2 | 0.3 |
| Do you need information about your implant or your convalescence? | 0.56 (0.22, 1.36) | 0.2 | 0.15 (0.01, 0.90) | 0.081 | 0.3 |

**Table S7: Primary implantation or revision**

|  | **Primary implantation** | | **Revision** | |  |
| --- | --- | --- | --- | --- | --- |
|  | **Odds Ratio (95% CI)** | **P** | **Odds Ratio (95% CI)** | **P** | **P for interaction** |
| Correct answer: manufacturer/brand or model (from memory) | 6.51 (1.93, 33.7) | **0.002** | 5.74 (0.42, 819) | 0.2 | 0.9 |
| Correct answer: manufacturer/brand or model (with the help of a list among those who failed from memory) | 1.97 (0.79, 5.20) | 0.2 | 1.71 (0.37, 8.29) | 0.5 | 0.9 |
| Correct answer: manufacturer/brand or model (from memory or with the help of a list) | 3.27 (1.53, 7.49) | **0.003** | 2.29 (0.53, 10.6) | 0.3 | 0.7 |
| Correct answer: establishment of the prosthesis placement | 1.01 (0.12, 8.55) | 0.9 | 2.15 (0.18, 49.7) | 0.6 | 0.6 |
| Correct answer: regular medical follow-up required? | 2.64 (1.45, 4.90) | **0.002** | 7.00 (0.94, 145) | 0.10 | 0.4 |
| Correct answer: prosthesis materials | 6.60 (3.61, 12.4) | **<0.001** | 4.00 (0.92, 19.9) | 0.074 | 0.5 |
| Correct answer: did you receive the patient-implant card (BI only) | 0.30 (0.00, 6.37) | 0.5 | 1.12 (0.01, 218) | 0.9 | 0.6 |
| Correct answer: did you receive the patient-implant form (THR/TKR only) | 2.67 (1.27, 5.86) | **0.011** | 3.75 (0.33, 93.9) | 0.3 | 0.8 |
| Correct answer: did you receive the patient-implant card or form (pooled) | 2.37 (1.16, 5.06) | **0.021** | 3.50 (0.39, 76.1) | 0.3 | 0.8 |
| Correct answer: date of prosthesis placement | 1.34 (0.64, 2.85) | 0.4 | 0.62 (0.07, 4.34) | 0.6 | 0.5 |
| Correct answer: surgeon’s name | 0.92 (0.39, 2.14) | 0.9 | 0.42 (0.05, 2.61) | 0.4 | 0.5 |
| Correct answer: prosthesis batch number (THR/TKR) | 1.11 (0.62, 2.00) | 0.7 | 0.75 (0.08, 6.93) | 0.8 | 0.7 |
| Correct answer: breast implant serial number | 1.00 (0.14, 6.92) | 0.9 | 0.50 (0.02, 6.45) | 0.6 | 0.7 |
| Correct answer: batch or serial number (THR/TKR or BI) | 1.10 (0.63, 1.92) | 0.7 | 0.73 (0.14, 3.51) | 0.7 | 0.6 |
| Correct answer to the 3 items: date of prosthesis placement, surgeon’s name, batch or serial number | 1.19 (0.69, 2.07) | 0.5 | 0.58 (0.13, 2.46) | 0.5 | 0.4 |
| Correct answer: texturing of your prosthesis surface (BI only) | 4.67 (0.82, 32.8) | 0.095 | 5.83 (0.78, 61.7) | 0.10 | 0.9 |
| Correct answer: scar at the front or at the back of the pelvis? (THR only) | 10.2 (1.05, 1,367 | **0.044** | 1.67 (0.06, 283) | 0.8 | 0.5 |
| Correct answer: fixed prosthesis with or without cement? (THR only) | 1.99 (0.95, 4.27) | **0.072** | 1.50 (0.04, 57.1) | **0.8** | 0.9 |
| Knowledge of the possibility of self-reporting an adverse event associated with the prosthesis | 2.37 (1.31, 4.34) | **0.005** | 5.09 (0.64, 107) | 0.2 | 0.5 |
| Correct answer: Who can you report a problem with your prosthesis to? | 1.00 (0.00, Inf) | 0.9 | 1.00 (0.00, Inf) | 0.9 | 0.9 |
| Level of confidence in the prosthesis (categorical from Lickert scale) | 0.82 (0.33, 2.00) | 0.7 | 4.33 (0.79, 34.5) | 0.11 | 0.11 |
| Call to the care unit where the patient was hospitalized after the prosthesis was placed | 3.16 (1.25, 9.10) | **0.021** | 3.25 (0.57, 26.2) | 0.2 | 0.9 |
| Consultation related to the prosthesis with a surgeon outside of the surgery centre | 0.44 (0.11, 1.54) | 0.2 | 1.00 (0.01, 190) | 0.9 | 0.7 |
| Admission to the emergency room for a complication related to the prosthesis | 1.41 (0.44, 4.92) | 0.6 | 0.29 (0.01, 2.57) | 0.3 | 0.2 |
| Hospitalization for a complication related to the prosthesis | 1.72 (0.61, 5.23) | 0.3 | 0.14 (0.01, 1.07) | 0.10 | 0.053 |
| Satisfaction with the information provided about the prosthesis after the operation (categorical from Lickert scale) | 1.33 (0.57, 3.15) | 0.5 | 1.63 (0.23, 14.0) | 0.6 | 0.9 |
| Do you need information about your implant or your convalescence? | 0.47 (0.17, 1.18) | 0.12 | 0.29 (0.05, 1.36) | 0.13 | 0.6 |

**Table S8: Breast augmentation surgery vs. breast reconstruction surgery**

|  | **Breast augmentation surgery** | | **Breast reconstruction surgery** | |  |
| --- | --- | --- | --- | --- | --- |
|  | **Odds Ratio (95% CI)** | **P** | **Odds Ratio (95% CI)** | **P** | **P for interaction** |
| Correct answer: manufacturer/brand or model (from memory) | 4.33 (0.68, 48.5) | 0.12 | 11.7 (0.75, 1,744) | 0.082 | 0.6 |
| Correct answer: manufacturer/brand or model (with the help of a list among those who failed from memory) | 2.78 (0.38, 24.0) | 0.3 | 1.00 (0.09, 11.3) | 0.9 | 0.5 |
| Correct answer: manufacturer/brand or model (from memory or with the help of a list) | 4.58 (0.77, 32.7) | 0.10 | 2.00 (0.26, 19.8) | 0.5 | 0.6 |
| Correct answer: establishment of the prosthesis placement | 1.00 (0.00, Inf) | 0.9 | 1.00 (0.00, Inf) | 0.9 | 0.9 |
| Correct answer: regular medical follow-up required? | 0.75 (0.03, 9.18) | 0.8 | 2.14 (0.20, 50.7) | 0.6 | 0.6 |
| Correct answer: prosthesis materials | 7.36 (1.05, 87.4) | **0.044** | 82.3 (5.46, 13,282) | **<0.001** | 0.2 |
| Correct answer: did you receive the patient-implant card (BI only) | 0.47 (0.00, 9.91) | 0.6 | 0.62 (0.00, 121) | 0.8 | 0.9 |
| Correct answer: did you receive the patient-implant form (THR/TKR only) |  |  |  |  |  |
| Correct answer: did you receive the patient-implant card or form (pooled) | 0.47 (0.00, 9.91) | 0.6 | 0.62 (0.00, 121) | 0.8 | 0.9 |
| Correct answer: date of prosthesis placement | 0.17 (0.00, 2.13) | 0.2 | 1.08 (0.11, 14.2) | 0.9 | 0.3 |
| Correct answer: surgeon’s name | 1.10 (0.15, 7.15) | 0.9 | 0.17 (0.00, 3.85) | 0.3 | 0.3 |
| Correct answer: prosthesis batch number (THR/TKR) |  |  |  |  |  |
| Correct answer: breast implant serial number | 0.71 (0.08, 4.81) | 0.7 | 1.25 (0.09, 31.2) | 0.9 | 0.7 |
| Correct answer: batch or serial number (THR/TKR or BI) | 0.71 (0.08, 4.81) | 0.7 | 1.25 (0.09, 31.2) | 0.9 | 0.7 |
| Correct answer to the 3 items: date of prosthesis placement, surgeon’s name, batch or serial number | 1.07 (0.19, 5.88) | 0.9 | 0.50 (0.04, 5.46) | 0.6 | 0.6 |
| Correct answer: texturing of your prosthesis surface (BI only) | 3.13 (0.56, 19.8) | 0.2 | 8.00 (0.92, 105) | 0.076 | 0.5 |
| Correct answer: fixed prosthesis with or without cement? (THR only) |  |  |  |  |  |
| Knowledge of the possibility of self-reporting an adverse event associated with the prosthesis | 6.50 (0.67, 148) | 0.14 | 0.56 (0.02, 16.1) | 0.7 | 0.2 |
| Correct answer: Who can you report a problem with your prosthesis to? | 1.00 (0.00, Inf) | 0.9 | 1.00 (0.00, Inf) | 0.9 | 0.9 |
| Level of confidence in the prosthesis (categorical from Lickert scale) | 5.12 (0.24, 782) | 0.3 | 0.58 (0.04, 8.50) | 0.7 | 0.3 |
| Call to the care unit where the patient was hospitalized after the prosthesis was placed | 24.8 (2.36, 3,428) | **0.004** | 1.73 (0.12, 25.6) | 0.7 | 0.2 |
| Consultation related to the prosthesis with a surgeon outside of the surgery centre | 0.20 (0.00, 4.09) | 0.3 | 1.62 (0.01, 317) | 0.8 | 0.4 |
| Admission to the emergency room for a complication related to the prosthesis | 0.20 (0.00, 4.09) | 0.3 | 1.62 (0.01, 317) | 0.8 | 0.4 |
| Hospitalization for a complication related to the prosthesis | 0.20 (0.00, 4.09) | 0.3 | 0.49 (0.00, 10.7) | 0.7 | 0.7 |
| Satisfaction with the information provided about the prosthesis after the operation (categorical from Lickert scale) | 1.63 (0.06, 45.1) | 0.7 | 0.56 (0.02, 16.1) | 0.7 | 0.6 |
| Do you need information about your implant or your convalescence? | 0.13 (0.01, 0.88) | **0.047** | 0.25 (0.02, 2.22) | 0.2 | 0.7 |

**Table S9: Level of education (Elementary/Middle school vs. High school and higher)**

|  | **Elementary/Middle school** | | **High school and higher** | |  |
| --- | --- | --- | --- | --- | --- |
|  | **Odds Ratio (95% CI)** | **P** | **Odds Ratio (95% CI)** | **P** | **P for interaction** |
| Correct answer: manufacturer/brand or model (from memory) | 9.18 (0.94, 1,229) | 0.058 | 6.12 (1.73, 32.4) | **0.004** | 0.8 |
| Correct answer: manufacturer/brand or model (with the help of a list among those who failed from memory) | 1.50 (0.44, 5.41) | 0.5 | 2.09 (0.79, 5.76) | 0.14 | 0.7 |
| Correct answer: manufacturer/brand or model (from memory or with the help of a list) | 2.36 (0.79, 8.01) | 0.14 | 3.34 (1.48, 8.02) | **0.005** | 0.6 |
| Correct answer: establishment of the prosthesis placement | 1.06 (0.12, 9.14) | 0.9 | 1.97 (0.18, 43.0) | 0.6 | 0.7 |
| Correct answer: regular medical follow-up required? | 5.47 (2.32, 13.8) | **<0.001** | 1.66 (0.76, 3.71) | 0.2 | **0.049** |
| Correct answer: prosthesis materials | 6.85 (2.80, 18.3) | **<0.001** | 6.71 (3.18, 14.8) | **<0.001** | 0.9 |
| Correct answer: did you receive the patient-implant card (BI only) | 3.00 (0.01, 703) | 0.6 | 0.28 (0.00, 5.62) | 0.4 | 0.4 |
| Correct answer: did you receive the patient-implant form (THR/TKR only) | 2.87 (1.04, 8.82) | 0.050 | 2.65 (1.00, 7.62) | 0.058 | 0.9 |
| Correct answer: did you receive the patient-implant card or form (pooled) | 3.05 (1.11, 9.33) | **0.038** | 2.06 (0.82, 5.48) | 0.13 | 0.6 |
| Correct answer: date of prosthesis placement | 2.08 (0.76, 6.07) | 0.2 | 0.76 (0.29, 1.98) | 0.6 | 0.2 |
| Correct answer: surgeon’s name | 0.73 (0.20, 2.45) | 0.6 | 0.85 (0.32, 2.27) | 0.7 | 0.8 |
| Correct answer: prosthesis batch number (THR/TKR) | 0.92 (0.42, 2.04) | 0.8 | 1.30 (0.57, 2.97) | 0.5 | 0.6 |
| Correct answer: breast implant serial number | 3.00 (0.01, 703) | 0.6 | 0.65 (0.14, 2.82) | 0.6 | 0.5 |
| Correct answer: batch or serial number (THR/TKR or BI) | 1.03 (0.48, 2.2) | 0.9 | 1.09 (0.53, 2.24) | 0.8 | 0.9 |
| Correct answer to the 3 items: date of prosthesis placement, surgeon’s name, batch or serial number | 1.13 (0.52, 2.46) | 0.8 | 1.07 (0.54, 2.15) | 0.8 | 0.9 |
| Correct answer: texturing of your prosthesis surface (BI only) | 0.33 (0.00, 10.1) | 0.5 | 6.82 (1.68, 32.6) | **0.007** | 0.11 |
| Correct answer: scar at the front or at the back of the pelvis? (THR only) | 1.04 (0.01, 194) | 0.9 | 12.6 (1.33, 1,682) | **0.023** | 0.3 |
| Correct answer: fixed prosthesis with or without cement? (THR only) | 2.33 (0.79, 7.23) | 0.13 | 1.79 (0.66, 4.98) | 0.3 | 0.7 |
| Knowledge of the possibility of self-reporting an adverse event associated with the prosthesis | 2.57 (1.14, 5.98) | **0.025** | 2.47 (1.12, 5.64) | **0.027** | 0.9 |
| Correct answer: Who can you report a problem with your prosthesis to? | 1.00 (0.00, Inf) | 0.9 | 1.00 (0.00, Inf) | 0.9 | 0.9 |
| Level of confidence in the prosthesis (categorical from Lickert scale) | 2.27 (0.56, 11.3) | 0.3 | 0.86 (0.33, 2.20) | 0.8 | 0.3 |
| Call to the care unit where the patient was hospitalized after the prosthesis was placed | 2.38 (0.62, 11.6) | 0.2 | 3.72 (1.34, 12.1) | **0.017** | 0.6 |
| Consultation related to the prosthesis with a surgeon outside of the surgery centre | 2.29 (0.36, 24.2) | 0.4 | 0.07 (0.00, 0.63) | **0.013** | **0.019** |
| Admission to the emergency room for a complication related to the prosthesis | 2.94 (0.36, 60.6) | 0.4 | 0.71 (0.20, 2.36) | 0.6 | 0.3 |
| Hospitalization for a complication related to the prosthesis | 2.38 (0.62, 11.6) | 0.2 | 0.48 (0.12, 1.62) | 0.3 | 0.10 |
| Satisfaction with the information provided about the prosthesis after the operation (categorical from Lickert scale) | 1.56 (0.46, 5.6) | 0.5 | 1.25 (0.46, 3.49) | 0.7 | 0.8 |
| Do you need information about your implant or your convalescence? | 0.42 (0.09, 1.61) | 0.2 | 0.43 (0.15, 1.10) | 0.087 | 0.9 |

**Table S10: Experience as a health care professional (no vs. yes)**

|  | **No** | | **Yes** | |  |
| --- | --- | --- | --- | --- | --- |
|  | **Odds Ratio (95% CI)** | **P** | **Odds Ratio (95% CI)** | **P** | **P for interaction** |
| Correct answer: manufacturer/brand or model (from memory) | 7.06 (2.11, 36.4) | **<0.001** | 3.18 (0.16, 478) | 0.5 | 0.7 |
| Correct answer: manufacturer/brand or model (with the help of a list among those who failed from memory) | 1.27 (0.56, 2.86) | 0.6 | 16.7 (1.64, 2,278) | **0.014** | **0.040** |
| Correct answer: manufacturer/brand or model (from memory or with the help of a list) | 2.24 (1.14, 4.56) | **0.019** | 19.8 (2.01, 2,674) | **0.007** | 0.082 |
| Correct answer: establishment of the prosthesis placement | 1.31 (0.31, 6.01) | 0.7 | 1.00 (0.01, 190) | 0.9 | 0.9 |
| Correct answer: regular medical follow-up required? | 2.82 (1.54, 5.29) | **<0.001** | 3.12 (0.56, 24.6) | 0.2 | 0.9 |
| Correct answer: prosthesis materials | 6.40 (3.48, 12.2) | **<0.001** | 6.67 (1.50, 37.7) | **0.018** | 0.9 |
| Correct answer: did you receive the patient-implant card (BI only) | 0.78 (0.00, 149) | 0.9 | 1.22 (0.01, 42.8) | 0.9 | 0.9 |
| Correct answer: did you receive the patient-implant form (THR/TKR only) | 3.00 (1.36, 7.05) | **0.008** | 1.60 (0.31, 9.53) | 0.6 | 0.5 |
| Correct answer: did you receive the patient-implant card or form (pooled) | 2.72 (1.25, 6.31) | **0.015** | 1.77 (0.40, 8.52) | 0.5 | 0.6 |
| Correct answer: date of prosthesis placement | 1.34 (0.64, 2.86) | 0.4 | 0.62 (0.07, 4.30) | 0.6 | 0.5 |
| Correct answer: surgeon’s name | 0.87 (0.40, 1.87) | 0.7 | 0.31 (0.00, 6.33) | 0.5 | 0.5 |
| Correct answer: prosthesis batch number (THR/TKR) | 1.29 (0.70, 2.37) | 0.4 | 0.40 (0.07, 2.04) | 0.3 | 0.2 |
| Correct answer: breast implant serial number | 1.02 (0.20, 5.41) | 0.9 | 0.60 (0.00, 16.8) | 0.8 | 0.8 |
| Correct answer: batch or serial number (THR/TKR or BI) | 1.20 (0.68, 2.10) | 0.5 | 0.44 (0.09, 1.88) | 0.3 | 0.2 |
| Correct answer to the 3 items: date of prosthesis placement, surgeon’s name, batch or serial number | 1.24 (0.72, 2.17) | 0.4 | 0.47 (0.11, 1.89) | 0.3 | 0.2 |
| Correct answer: texturing of your prosthesis surface (BI only) | 4.49 (1.10, 21.0) | **0.036** | 5.40 (0.20, 924) | 0.3 | 0.9 |
| Correct answer: scar at the front or at the back of the pelvis? (THR only) | 11.9 (1.29, 1,579) | **0.025** | 1.13 (0.01, 222) | 0.9 | 0.4 |
| Correct answer: fixed prosthesis with or without cement? (THR only) | 1.64 (0.76, 3.59) | 0.2 | 10.0 (1.01, 246) | 0.077 | 0.2 |
| Knowledge of the possibility of self-reporting an adverse event associated with the prosthesis | 1.97 (1.07, 3.66) | **0.030** | 10.7 (2.13, 83.2) | **0.008** | 0.075 |
| Correct answer: Who can you report a problem with your prosthesis to? | 1.00 (0.00, Inf) | 0.9 | 1.00 (0.00, Inf) | 0.9 | 0.9 |
| Level of confidence in the prosthesis (categorical from Lickert scale) | 1.44 (0.63, 3.39) | 0.4 | 0.29 (0.01, 2.58) | 0.3 | 0.2 |
| Call to the care unit where the patient was hospitalized after the prosthesis was placed | 2.25 (0.97, 5.63) | 0.060 | 15.4 (1.52, 2,092) | **0.017** | 0.15 |
| Consultation related to the prosthesis with a surgeon outside of the surgery centre | 0.52 (0.12, 1.88) | 0.3 | 0.31 0.00, 6.33 | 0.5 | 0.8 |
| Admission to the emergency room for a complication related to the prosthesis | 0.74 (0.25, 2.13) | 0.6 | 5.65 (0.42, 802) | 0.2 | 0.2 |
| Hospitalization for a complication related to the prosthesis | 0.99 (0.37, 2.64) | 0.9 | 1.00 (0.11, 9.24) | 0.9 | 0.9 |
| Satisfaction with the information provided about the prosthesis after the operation (categorical from Lickert scale) | 1.33 (0.57, 3.16) | 0.5 | 1.61 (0.23, 13.6) | 0.6 | 0.9 |
| Do you need information about your implant or your convalescence? | 0.49 (0.20, 1.13) | 0.10 | 0.20 (0.01, 1.58) | 0.2 | 0.5 |
